# Pheromone‐binding proteins based phylogenetics and phylogeography of *Maruca* spp. from Asia, Africa, Oceania, and South America

**DOI:** 10.1002/ece3.5471

**Published:** 2019-07-31

**Authors:** Periasamy Malini, Srinivasan Ramasamy, Roland Schafleitner, Krishnan Muthukalingan

**Affiliations:** ^1^ World Vegetable Center Shanhua Tainan Taiwan; ^2^ Bharathidasan University Tiruchirappalli Tamil Nadu India; ^3^ Present address: Madurai Kamaraj University Madurai Tamil Nadu India

**Keywords:** Automatic Barcode Gap Discovery, haplotype, haplotype network, Maruca, pheromone‐binding protein, phylogenetic analysis

## Abstract

Variations in the functional response of legume pod borer (*Maruca vitrata*) populations to sex pheromone blends were observed in Asia and Africa. Hence, this study was carried out to understand the differences in pheromone‐binding proteins (PBPs) among *Maruca* populations in Asia, Africa, Oceania, and South America. A de novo transcriptome assembly was adopted to sequence the entire transcribed mRNAs in *M. vitrata* from Taiwan. The raw‐sequence data were assembled using homologous genes from related organisms in GenBank to detect *M. vitrata* PBPs (MvitPBPs). Sections of the cDNA of *MvitPBP* of different length were used to design primers to amplify the full‐length cDNA of PBPs. All three PBP sequences comprised three exons interspersed by two introns. In total, 92 *MvitPBP1* haplotypes, 77 *MvitPBP2* haplotypes, and 64 *MvitPBP3* haplotypes were identified in 105, 98, and 68 *Maruca* individuals, respectively. High pairwise *F*_ST_ values (0.41–0.73) and phylogenetic analyses distinguished the putative *Maruca* species in South America from those occurring in rest of the world, and possibly two putative subspecies in Asia and Africa. The haplotype networks and Automatic Barcode Gap Discovery analyses also confirmed these results. The negative Tajima's *D* and Fu's *F*_S_ values showed the recent demographic expansion of *Maruca* populations. Thus, this study confirmed the presence of different *Maruca* species and/or subspecies in different continents based on the diversity within PBP genes. Additional sampling and studies are suggested for Oceania and South America. The genetic differences among *Maruca* populations should be carefully considered while using sex pheromone lures and bio‐control agents.

## 1 INTRODUCTION

Legume pod borer, *Maruca vitrata* (F.) (syn. *M*. *testulalis*) (Lepidoptera: Crambidae), is a major pest of food legumes in Asia, Africa, Americas, and Oceania (Malini, Srinivasan, Lin, Yule, & Krishnan, 2014; Sharma, 1998). It causes extensive damage to the flowers and pods. For example, 36% flower and pod damage due to *M. vitrata* infestation occurred in cowpea in Thailand (Phompanjai & Jamjanya, 2000). Grain yield losses of 50%–71% were reported in pigeon pea and Adzuki bean (Sharma & Franzmann, 2000). About 20%–30% pod damage in mung bean in Bangladesh (Zahid, Islam, & Begum, 2008) and 25% pod damage in yard‐long bean due to *M. vitrata* in west Sumatra (Hammig, Shepard, Carner, Dilts, & Rauf, 2008) were reported. Up to 380,000 t of cowpea was lost due to *M. vitrata* in Malawi, Senegal, Niger, Tanzania, and Kenya (Gressel et al., 2004). In Brazil, *M. vitrata* is considered as a seasonal pest on soybean (Hoffmann‐Campo et al., 2000), and it caused about 56% damage (Grigolli, Lourenção, & Ávila, 2015). *M. vitrata* caused more than 65% grain yield reduction in pigeon pea in Australia (Sharma, Saxena, & Bhagwat, 1999). Hence, farmers rely more on chemical pesticides to combat this pest. For instance, more than 80% of the yard‐long bean growers in Cambodia, Lao PDR (Laos), Thailand and Vietnam predominantly relied on synthetic pesticides (Schreinemachers et al., 2017, 2014). On an average, Thai yard‐long bean growers used 16.3 kg/ha of pesticide formulations per cropping cycle (Schreinemachers et al., 2014), and Cambodian farmers mixed four pesticides together in a single spray (Schreinemachers et al., 2017). Such an intensive pesticide use has serious consequences on human and environmental health. Hence, alternative pest management strategies are warranted for legume growers.

Insect pheromones are an important component in pest management programs, especially as a monitoring, mating‐disruption, and/or mass‐trapping tool. *M. vitrata* sex pheromone consists of one major and two minor compounds (Adati & Tatsuki, 1999; Downham et al., 2003). A synthetic sex pheromone consisting of major [(E,E)‐10,12‐hexadecadienal] and minor [(E,E)‐10,12‐hexadecadienol and (E)‐10‐hexadecenal] compounds developed in a ratio of 100:5:5 attracted male moths in Benin and Ghana, whereas the major compound alone was most effective in Burkina Faso (Downham et al., 2003, 2004). However, none of these blends attracted any males in Taiwan (Schläger et al., 2012), Thailand, and Vietnam (Srinivasan et al., 2015), although a variant blend was attractive in India (Hassan, 2007). These differential responses suggest the presence of genetically different *M. vitrata* populations.

An earlier study showed evidence for the presence of multiple *Maruca* species or subspecies (Margam et al., 2011). Herbison‐Evans, Hacobian, and Crossley (2017) also reported two forms of *M. vitrata* in Australia. However, we undertook a detailed study investigating the mitochondrial cytochrome c oxidase I (*coxI*) diversity in populations from Southeast Asia (the probable center of origin for *Maruca*), South Asia, sub‐Saharan Africa, and in reference populations from Oceania and Latin America. This study confirmed the presence of three putative *Maruca* species, including one in Latin America, one in Oceania (including Indonesia) and *M. vitrata* in Asia, Africa and Oceania (Malini, Schafleitner, Muthukalingan, & Ramasamy, 2015). The results also showed the presence of two putative *M. vitrata* subspecies in Asia and Africa.

Since different species or subspecies seem to exist in the genus *Maruca*, the pheromone composition and their reception may not be uniform in different geographical locations. A recent study found only two pheromone compounds in *M. vitrata* populations from Taiwan, Thailand, Vietnam, and Benin (Schläger et al., 2015). Similarly, different *M. vitrata* populations also produce pheromone compounds in different ratio. *M. vitrata* females from Wuhan and Huazhou provinces in China produced different ratio of the three compound pheromones (Lu, Qiao, & Luo, 2013). Thus, the pheromone composition in *M. vitrata* seems to vary across locations. Hence, it has been hypothesized that variations in the *M. vitrata* male pheromone reception may be attributed to the presence of different pheromone strains in *M. vitrata* females.

Insect sex pheromones facilitate the mate‐finding among the members of an insect species. In male moths, a specialized subset of chemosensilla contains pheromone‐sensitive neurons, which are highly sensitive and specific to sex pheromone compounds produced by conspecific females (LaForest, Prestwich, & Löfstedt, 1999). At the molecular level, the reception of pheromones in male moths is mediated by pheromone‐binding proteins (PBPs), a subfamily of odorant‐binding proteins (OBPs). PBPs which are localized in the lymph of the sensilla surrounding the olfactory neuron cells on the moth antennae (Vogt, Rogers, Franco, & Sun, 2002) bind to the lipophilic pheromonal compounds (Bette, Breer, & Krieger, 2002; Lautenschlager, Leal, & Clardy, 2007; Maida, Ziegelberger, & Kaissling, 2003; Steinbrecht, Laue, & Ziegelberger, 1995; Vogt & Riddiford, 1981) and carry them to the receptor cells (Van den Berg & Ziegelberger, 1991). It has been demonstrated that the change in male pheromone response behavior is caused by differences in a sex‐linked locus or set of linked loci (Willett & Harrison, 1999). The gene loci that are instrumental in conferring specificity in pheromone communication systems should show fixed amino acid differences between strains or species (Willett & Harrison, 1999). Thus, understanding the patterns of variation in the gene encoding PBP could provide insights into the population structure of *Maruca* spp., which differed in their responses to the same pheromone blend(s) in different geographical locations. Hence, this study was carried out to assess whether there are fixed nucleotide differences at the PBP locus between the pheromone strains of *Maruca* from different host plants and geographical origin.

## 2 MATERIALS AND METHODS

### 2.1 Insects

A *Maruca vitrata* colony was established at the Insectary of World Vegetable Center from a field population. The larvae were reared on *Spodoptera exigua* meridic diet (Bio‐Serv, Frenchtown, NJ, USA) modified with cowpea powder, at 27 ± 1°C and 70 ± 10% relative humidity, photoperiod 14:10 hr (Light:Dark) until pupation. On pupation, they were sexed and placed in acrylic cylinders (30‐cm long and 15‐cm diameter), whose ends were covered with nylon‐nets. Emerged adults were fed with 10% (w/v) sugar solution. Besides from Taiwan, *M. vitrata* larval populations from nine countries (Bangladesh, Benin, Indonesia, India, Kenya, Laos, Malaysia, Thailand, and Vietnam) from different host plants were collected (Malini et al., 2015). Additional *Maruca* larval samples were collected from nine host plants (*Dioclea* sp., *Dioclea guianensis*, *Dioclea trujellensis*, *Phaseolus vulgaris*, *Vigna unguiculata*. subsp. *sesquipedalis*, *Lablab purpureus*, *Psophocarpus tetragonolobus*, *Tephrosia candida*, and *Pueraria phaseoloides*) in five countries [Cambodia (11°30′41.3″N 105°02′30.6″E; 11°51′39.6″N 105°01′41.1″E), Colombia (03°03′27.1″N 76°29′42.1″W; 03°13′53.7″N 76°13′54.6″W; 03°30′09.3″N 76°21′26.0″W), Fiji (Sigatoka Valley), Indonesia (East Kalimantan), and Papua New Guinea (PNG) (06°00′48.8″S 145°19′18.6″E to 06°22′00.4″S 145°54′29.6″E; 05°51′10.6″S 145°43′56.5″E; 05°51′20.2″S 145°41′53.5″E; 05°35′53.9″S 145°27′40.5″E; 06°41′19.1″S 146°51′04.1″E; 06°43′24.5″S 146°46′46.6″E; 10°18′37.6″S 150°20′02.0″E; 10°20′18.0″S 150°38′33.7″E)]. The collected larval samples were preserved in 95% ethanol. The Asian and African *Maruca* samples for PBP studies were mostly chosen based on the *coxI* haplotypes in Malini et al. (2015).

### 2.2 RNA extraction, complementary DNA (cDNA) synthesis and reverse transcription polymerase chain reaction (RT‐PCR) amplification

About 100 antennae were used to obtain about 25 mg of the tissues. Total RNA was isolated from homogenized tissue using the RNeasy kit (Qiagen) following the manufacturer's protocol, with in‐column DNase I treatment. RNA was quantified spectroscopically at 260‐nm, and purity was estimated using a Spectrophotometer and assayed for purity based on the A_260_/A_280_ ratio. cDNA was synthesized from total RNA using SuperScript III Reverse Transcriptase, RNaseOUT (Invitrogen), and a mixture of random hexamer and oligo (dT)_20_ primers following the manufacturers’ protocols. Eight micro liter of total RNA (190 ng) was mixed with 1 μl of 50 μM oligo (dT)_20_ and 4 μl of 2.5 mM dNTP mix. This mixture was incubated at 65°C for 5 min and then stored on ice. The RT‐PCR mix was prepared by mixing 4 μl of 5X First‐Strand Buffer, 1 μl of 0.1 M DTT, 1 μl of RNaseOUT (40 units/μl), and 1 μl of SuperScript III Reverse Transcriptase (200 units/μl). The mix was added to the RNA solution and centrifuged briefly. Reverse Transcription was performed at 50°C for 60 min and stopped by heating the reaction mixture to 70°C for 5 min.

Reverse transcription polymerase chain reaction amplification was performed in a total reaction volume of 25 μl containing 120–180 ng of first‐strand cDNA, 10X PCR Gold Buffer, 0.5 μM of each primer (Table 1), 2.5 mM MgCl_2_, 0.2 mM dNTP, and 0.04 unit/μl of Super‐Therm Gold DNA Polymerase (Bertec Enterprise, Taipei, Taiwan). Cycling conditions were as follows: initial denaturation at 95°C for 10 min; 36 cycles of 94°C for 1 min, 60°C for 50 s, and 72°C for 1 min; and final extension at 72°C for 10 min. The RT‐PCR products were visualized after 1% agarose gel electrophoresis and ethidium bromide staining under UV light.

**Table 1:** Oligonucleotide primers designed and used for isolation and identification of *M. vitrata* pheromone‐binding protein (PBP) genes

Gene	Primer type	Forward primer	Reverse primer	PCR product range (kb)
PBP1	Main primer	5′‐CAGGAGCTGAAAATGGAGTTG‐3′	5′‐CTAGACGTGGGCTGTCCTTC‐3′	1.2–2.3
	Alternative primer	5′‐GTTGCAGGAGCTGAAAATGG‐3′	5′‐GCTGTCCTTCGGGTAACATC‐3′	
	Internal primer	5′‐CTC ATC TGC ATG TCC ACC A ‐3′	5′‐CTT GGT GGA CAT CCA GAT GAG‐3′	
PBP2	Main primer	5′‐AATGGCCTAAAGGGCCACAA‐3′	5′‐AGGTTTCATGTCACAATCTTCATC‐3′	1.1–3.0
	Alternative primer	5′‐CTAAAGGGCCACAAACTTAACC‐3′	5′‐TAAGTACTCTTGCGAAGCCGAA‐3′	
	Internal primer	5′‐TAC GAG GTC AAA ACT TCG AGA AG‐3′	5′‐CGC TTC TCG ACT TTT GAC CT‐3′	
PBP3	Main primer	5′‐GCATACAGTTTCCGTTTTCATCC‐3′	5′‐GGAGGTCCTTTCGTTCAGACTT‐3′	1.2–2.1
	Alternative primer	5′‐AACGCGCAAAGTAAACGAAC‐3′	5′‐ACTTCAGCCAGCATCTCTCC‐3′	
	Internal primer	5′‐CAG GAG GTG ATG ACC AAA ATG AG‐3′	5′‐TTG TAA GCG TTC TCG TGG TG‐3′	

### 2.3 DNA extraction

The total DNA was extracted from individual larva of *Maruca* using three methods: (A) using Easy DNA High‐speed Extraction Tissue Kit (Saturn Biotech); (B) using BuccalAmp DNA Extraction Kit (Bio‐Genesis Technologies) for the populations from Asia and Africa, and additional details were provided in Malini et al. (2015). Third method used was gSYNC^™^ DNA Extraction Kit (Geneaid) for populations from Oceania and South America. The DNA solution was treated with RNase and Proteinase K, and stored in aliquots at −20°C.

### 2.4 Sequencing the genes encoding pheromone‐binding proteins

#### 2.4.1 Amplification of PBP using gene‐specific primers

PCR primers specific for PBP genes were designed based on the *M. vitrata* transcriptome sequence (Chang & Srinivasan, 2014) using Primer3 (Untergasser et al., 2007), and their quality was checked in PCR Primer Stats (http://www.bioinformatics.org/sms2/pcr_primer_stats.html). These primers were used in the RT‐PCR to confirm the PBP genes as well as primer specificity, and they were used for genomic DNA analysis of various *Maruca* populations. The main primers (Table 1) located at the 5′ and 3′ untranslated regions (UTRs) were expected to amplify the full‐length sequence of the PBP genes. An alternate primer pair for each PBP was designed for those samples which failed to amplify. As PBP genes are relatively long (≈1,200–2,600 bp) because of introns, internal primers were also designed and used to obtain the full‐length sequences of the target PBPs. Gradient PCR was performed to determine the optimal annealing temperatures for these primers.

#### 2.4.2 Polymerase chain reaction amplification of PBP

The PCR amplification was performed in 25 μl reaction volume containing 80–120 ng of genomic DNA. The remaining content of the PCR mixture was the same as described in 2.2. PCR was performed in a MJ Research Thermocycler (PTC200 DNA Engine Cycler, Bio‐Rad Laboratories, Inc.). Annealing temperatures were 48–72°C (PBP1), 48–69°C (PBP2) and 48–70°C (PBP3). Those samples which failed to yield amplification products with the above PCR conditions were amplified using a touch‐down PCR with nine cycles of 94°C for 50 s, 50° to 66°C for 1 min (–0.5°C per cycle), and 72°C for 30 s; 25 cycles of 94°C for 50 s, 55°C for 1 min, and 75°C for 30 s. The PCR products were visualized on 1% agarose gels and ethidium bromide staining under UV light and sequenced at Genomics BioSci & Tech. Co., Ltd, Taiwan. In case of multiple amplification products, single bands were extracted from the agarose gels using Geneaid extraction kit.

### 2.5 Molecular divergence and population genetic analyses

The *MvitPBP1*, *MvitPBP2*, and *MvitPBP3* sequences were aligned and edited using BioEdit v7.0 (Hall, 1999). To determine introns and intron‐exon boundaries, the *MvitPBP* genomic DNA sequences were subject to ClustalW analysis against the corresponding cDNA sequence of *M. vitrata* transcriptome. After removing the introns and UTRs, the obtained sequences were used to find the signal peptide using SignalP‐5.0 Server and were examined for polymorphisms in the coding regions of the *MvitPBP* genes among *Maruca* populations. Since we obtained shorter 5′‐UTR for *PBP1* from our transcriptome sequence, we were unable to obtain clear sequence for the signal peptide for some of the populations. Hence, the signal peptide of *PBP1* was not included for the analysis, but the ORF was used for *MvitPBP2* and *MvitPBP3* analyses. The number of haplotypes, nucleotide diversity, and haplotype diversity were calculated for investigating the *PBP* sequence diversity using DnaSP 5.10 (Librado & Rozas, 2009). Statistical tests of Tajima's *D* and Fu's *F_S_* values were used to detect the deviation from the neutral model of evolution using DnaSP 5.10. Tajima's *D* uses mutation frequencies in the sequences to identify if a population has undergone a recent population expansion event and is determined by the difference between average number of nucleotide differences and the number of segregating sites estimated from pairwise comparisons (Tajima, 1989). Fu's *F_S_* test uses information from the haplotype distribution in a sample. The test estimates the probability of observing a random sample with equal or less singletons than the observed given a level of diversity. The test is based on the infinite site mutation model and assumes that all of the alleles are selectively neutral.

The genetic structure of *M. vitrata* populations based on various *PBP* sequences was examined by analysis of molecular variance (AMOVA) using Arlequin 2.001 (Schneider, Roessli, & Excoffier, 2000). This method was used to partition the genetic variance within and among populations as well as within and among groups. The populations were grouped by geographical locations (continents). Levels of significance were determined through 1,000 random permutation replicates. Pairwise *F*_ST_ values used to appraise the genetic structure among populations were obtained with 1,000 permutations and at the significance level of 0.05 using the K2P model (Kimura, 1980).

### 2.6 Phylogenetic, species delineation, and haplotype network analyses

The FASTA formatted coding regions of *MvitPBP* sequences were imported into the MEGA‐X software package sequence alignment application, and a multiple sequence alignment was performed with the ClustalW algorithm using default parameters (Tamura et al., 2011). The aligned sequences were used for phylogenetic analysis. The evolutionary history among the haplotypes of *MvitPBP* sequences was inferred by using the maximum likelihood method in MEGA‐X (Kumar, Stecher, Li, Knyaz, & Tamura, 2018). The appropriate model of sequence evolution, including model parameters, was calculated using corrected Akaike Information Criterion and resulted in T92 + G+I (Tamura 3‐parameter using a discrete Gamma distribution plus assuming that a certain fraction of sites is evolutionarily invariable) (Tamura, 1992) as the best model for *MvitPBP1*. The best model for *MvitPBP2* was K2 (Kimura 2‐parameter)+G + I, whereas K2 + G was selected for *MvitPBP3*. The models were also selected based on partitioning by codon position. Initial tree(s) for the heuristic search were obtained automatically by applying Neighbor‐Joining and BioNJ algorithms to a matrix of pairwise distances estimated using the maximum composite likelihood approach, and then selecting the topology with superior log likelihood value. The bootstrap consensus tree inferred from 1,000 replicates (Felsenstein, 1985) was taken to represent the evolutionary history. Branches corresponding to partitions reproduced in less than 50% of the bootstrap replicates were collapsed. The percentage of replicate trees in which the samples clustered together in the bootstrap test is shown next to the branches (Felsenstein, 1985). The phylogenetic trees were rooted by the outgroup *Conogethes punctiferalis*.

The primary species hypothesis was evaluated using Automatic Barcode Gap Discovery (ABGD), a molecular species delineation method. ABGD is an automated procedure that clusters sequences into candidate species based on pairwise distances by detecting differences between intra‐ and interspecific variation without a priori species hypothesis (Puillandre, Lambert, Brouillet, & Achaz, 2012). The program requires a prior limit to intraspecific diversity (P) and a proxy for minimum gap width (X). *MvitPBP* sequences were analyzed in the web‐server of ABGD (http://wwwabi.snv.jussieu.fr/public/abgd/abgdweb.html) using the Jukes–Cantor (JC69) model, a gap width of 0.99 (for *MvitPBP1* and *MvitPBP2*) and 1.50 (for *MvitPBP3*) and the *p* value from .001 to .05. The genealogical relationships among *M. vitrata* *PBP* sequences were also examined by establishing a TCS haplotype network with the software Population Analysis with Reticulate Trees (Clement, Posada, & Crandall, 2000).

## 3 RESULTS

### 3.1 Structure of *M. vitrata* PBP genes

**Figure 1:**
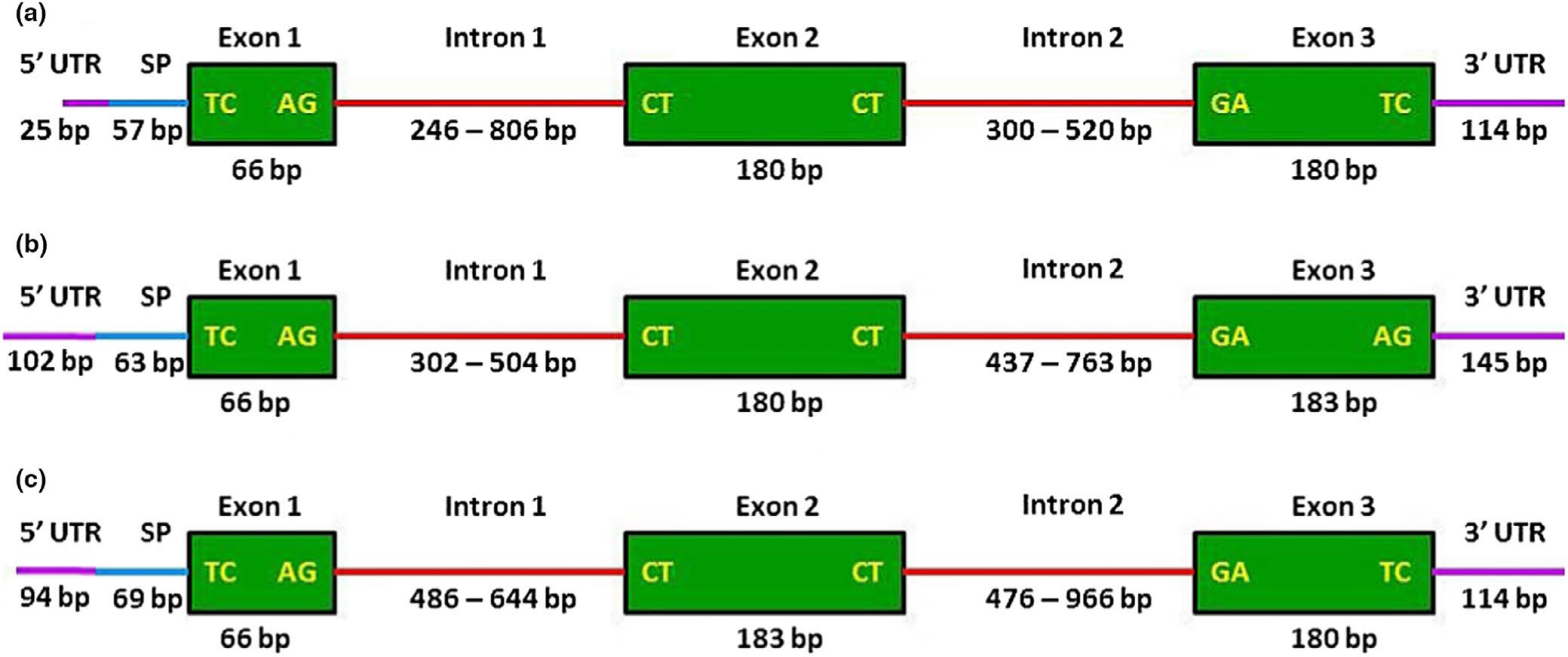
Structure of the (a) *MvitPBP1*, (b) *MvitPBP2*, and (c) *MvitPBP3* genomic DNA among *Maruca* populations from Asia, Africa, Oceania, and South America. SP indicates the signal peptide

The assembly of the candidate homologs from the transcriptome sequence of *M. vitrata* population from Taiwan matching to PBP of other closely related species resulted in unigenes of PBP1, PBP2, and PBP3, and deposited in the GenBank (IDs: AGS46557, AGS46556, and QDA95521), which have been designated as *MvitPBP1*, *MvitPBP2*, and *MvitPBP3*. The structure of the *MvitPBPs* is shown in Figure 1. The 626, 742, and 621 bp cDNA portions from *M. vitrata* used to design the primer pairs for PBP1, PBP2, and PBP3, respectively, amplified the full‐length sequences of PBPs in *Maruca* populations.

The *M. vitrata* PBP gene‐specific primers amplified PCR products of 1.1–3.0 kb size in *M. vitrata* populations from different continents (Table 1). The size of *MvitPBP1*, *MvitPBP2*, or *MvitPBP3* varied among the populations depending on the intron size. Few individuals of some populations produced more than one specific band. Upon gel‐purification and sequencing, it showed that the different band sizes (Table 2) were due to size differences of introns 1 and 2. The two forms also showed polymorphisms, mostly in exons 2 and 3 for all three PBPs. Based on the sequences of these two forms, they are not due to internal primer binding sites, but due to heterozygosity among the individuals of a population. We obtained a consensus sequence of 426 bp (without signal peptide) for *MvitPBP1*, 495 bp for *MvitPBP2*, and 501 bp for *MvitPBP3* across all *Maruca* populations.

**Table 2:** Size differences among the isoforms of *M. vitrata* pheromone‐binding protein (PBP) genes in selected individuals of different populations

Gene	Lower band (kb)	Upper band (kb)	Example (population)
*MvitPBP1*	≈1.5	≈2.0	Vietnam (VVB6B, VVB6T) Malaysia (IM1B, IM1T, IM2B, IM2T)
*MvitPBP2*	≈1.6	≈2.8	Thailand (VT1B, VT1T) Malaysia (VM3B, VM3T, OMK1B, OMK1T, OMK4B, OMK4T)
*MvitPBP3*	≈1.3	≈2.9	Laos (DL1B, DL1T) Thailand (MT3B, MT3T)

The varying length of introns of *MvitPBPs* is shown in Figure 1. Generally, African populations had longer introns than in other populations. For *MvitPBP2*, both introns were shorter in the African populations than in other populations. In both *MvitPBP2* and *MvitPBP3*, intron 2 was longer than intron 1, whereas intron 1 was longer than intron 2 in *MvitPBP1*.

### 3.2 Amino acid analysis of MvitPBP1, MvitPBP2, and MvitPBP3 and comparison to homologs of other related species

**Figure 2:**
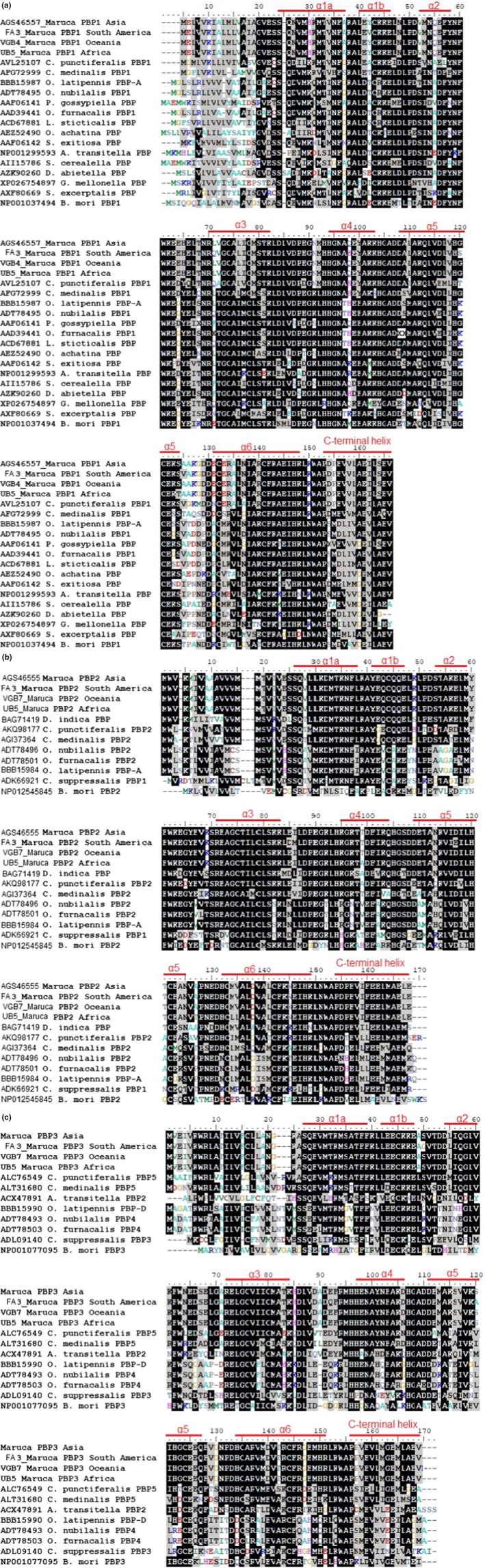
Multiple sequence alignment of MvitPBPs from Asia, Africa, Oceania, and South America with other Crambidae and Pyralidae moth as well as *Bombyx mori* PBPs. (a) MvitPBP1, (b) MvitPBP2, and (c) MvitPBP3. The red line indicates the α‐helices

The MvitPBP1, MvitPBP2, and MvitPBP3 contain 19, 21, and 23 amino acids, respectively, as signal peptides and 142, 143, and 143 amino acids in their mature proteins (Figure 1). The molecular mass of the predicted MvitPBP1, 2, and 3 proteins is 16.07 kDa, 16.36 kDa, and 16.31 kDa, respectively, which is typical for insect PBPs (16–18 kDa). MvitPBP1 protein contains more Leu, Glu, and Ala residues than other amino acids; MvitPBP2 contains more Leu, Glu, and Lys residues, while MvitPBP3 contains more Glu, Ala, and Val residues than other amino acids. The amino acid sequence analysis of MvitPBPs revealed that they consisted of seven α‐helices and a conserved motif of six cysteine residues. The location of the α‐helices has been predicted following Sandler, Nikonova, Leal, and Clardy (2000) to be located between residues 1–13 (α1a), 16–22 (α1b), 28–34 (α2), 46–58 (α3), 70–79 (α4), 84–100 (α5), and 107–124 (α6). The C‐terminal helix contains residues 129–142. The amino acid residues 60–69 form a loop, which is the flexible region of the protein. An alignment of the deduced amino acid sequences of MvitPBP1, MvitPBP2, and MvitPBP3, and other related species selected from Crambidae and Pyralidae is shown in Figure 2a–c. MvitPBP1 shared a moderate sequence identity with orthologs of *Conogethes punctiferalis*, *Cnaphalocrocis medinalis, Ostrinia* spp., and *Orthaga achatina*.

MvitPBP1 exhibited the highest similarity with CpunPBP1 of *C. punctiferalis* (87%), followed by CmedPBP1 of *C. medinalis* (86%). MvitPBP1 amino acid sequence also did not vary much with the PBPs or GOBPs of the members of insect families including Crambidae, Pyralidae, and Bombycidae (Figure 2a). There is no fixed variation in MvitPBP1 amino acid sequences among different *Maruca* populations from Asia, Africa, and Oceania. However, there are six specific amino acid substitutions in South America populations. Met10 in the signal peptide is replaced by Leu10. Tyr18, Leu47, Gln74, Gly96, and Ala101 of other *M. vitrata* populations were substituted by Val18, Gln47, His74, Ser96, and Val101, respectively.

The MvitPBP2 amino acid sequence shared high sequence similarity (85%–90%) with orthologs of *C. medinalis, C. punctiferalis,* and *Diaphania indica*. MvitPBP2 exhibited the most similarity (90%) to DindPBP (BAG71419) of *D. indica*. However, MvitPBP2 differed among the *M. vitrata* populations even at the amino acid level, and with the other species from Crambidae, Pyralidae, and Bombycidae (Figure 2b). Interestingly, Thr74 in other *Maruca* populations was substituted by Ala74 in all the African populations. Similarly, Ala102, Met109, and Val110 were substituted by Thr102 (except two samples), Leu109, and Leu110, respectively, in all African populations. Hence, these substitutions in positions 74, 102, 109, and 110 differentiate the African populations from other continental populations. In South American *Maruca* populations and a Fijian sample (VF4), Glu60, Leu61, and Asp92 (also for VF3) were substituted by Asp60, Met61, and Glu92, respectively. Unlike other MvitPBPs, few Asian and Oceania *M. vitrata* populations have Thr instead of Ala in the eighth position of signal peptide, while two Benin and a PNG populations have Met instead of Val in the 12th position; few African populations have Ala instead of Thr in the 16th position. Thus, MvitPBP2 possesses slight differences in its sequences.

The MvitPBP3 amino acid sequence shared high sequence similarity with orthologs of *C. punctiferalis* (73%) and *C. medinalis* (69%) (Figure 2c). MvitPBP3 differed by three amino acid substitutions in the South American populations compared to other populations. Ser24, Glu66, and Ser94 in all other *M. vitrata* populations were substituted by Gly24, Gln66, and Ala94, respectively, in the South American *Maruca* populations. Glu66 substitution in lieu of Gln66 was also found in one PNG population (VGG1). Asn80 in Africa *M. vitrata* populations was replaced by Asp80 in rest of the *Maruca* populations, except in one Vietnam population (BV1). Similarly, Lys21 in few Kenya *M. vitrata* populations was substituted by Thr21. Asp40 in most of the populations was substituted by Glu40 in one PNG (VGG5B) and one Laos (HL7) sample. Similar substitution was also found for one sample each from Indonesia (VNK8) and Benin (LB5) at position 65. One sample each from Malaysia (IMS5), PNG (EGW9), and Fiji (VF1) had Ser104 in lieu of Gly104 in all other populations.

### 3.3 PBPs haplotype variation in *M. vitrata* population and neutrality tests

The haplotypes identified in *Maruca* individuals were deposited in the NCBI GenBank (*MvitPBP1*: MK548942–MK549033, *MvitPBP2*: MK549034–MK549121, *MvitPBP3*: MK561786–MK561853) (Appendices 1–3). To a high extent, the haplotypes were specific to individual insects (80 for *MvitPBP1*, 62 each for *MvitPBP2* and *MvitPBP3*); only a small proportion (12, 15, and 2 for *MvitPBP1*, *2* and *3*, respectively) was present in multiple samples. Only one haplotype (Haplotype 43) was shared by three individuals from Indonesia and Thailand for *MvitPBP1* (Appendix 1). For *MvitPBP2*, the largest haplotype (Haplotype 2) contains five *Maruca* individuals, collected from Colombia and Fiji (Appendix 2). One haplotype (Haplotype 59) from Kenya shared four individuals for *MvitPBP3* (Appendix 3).

The total nucleotide diversity of all *Maruca* populations from sampled countries was 0.02391, 0.02507, and 0.02501 for *MvitPBP1*, *MvitPBP2,* and *MvitPBP3*, respectively (Table 3). In *MvitPBP1*, the nucleotide diversity of the *M. vitrata* populations from Thailand was the lowest and the one from Benin was the highest. In *MvitPBP2*, lowest nucleotide diversity was observed for *Maruca* populations from Colombia, whereas it was highest for Colombia based on *MvitPBP3*. The nucleotide diversity was almost similar for all other sampled countries in both *MvitPBP2* and *MvitPBP3*. Because of the large number of unique haplotypes, the haplotype diversity was one or close to one for most of the sampled countries for all the *MvitPBP* genes (Table 3). The lowest haplotype diversity was recorded for Colombia, only based on *MvitPBP1* and *MvitPBP2* genes.

**Table 3:** List of number of samples studied, number of haplotypes, haplotype diversity (*h*), nucleotide diversity (π), Tajima's *D* and Fu's *F_S_* tests for *Maruca* spp. populations from 12 countries in South and Southeast Asia, sub‐Saharan Africa, Oceania, and South America

Country	No. of samples	No. of haplotypes	Haplotype diversity (*h*)	Nucleotide diversity (π)	Tajima's *D*	Tajima's D (NonSyn/Syn) ratio	Fu's *F_S_*
*MvitPBP1*							
India (including Bangladesh)	4	4	1.000	0.01800	0.04025	–	0.017
Thailand	6	3	0.800	0.01002	1.28387	–	2.584
Cambodia	6	3	0.800	0.01753	1.33727	0.63881	3.996
Laos	7	7	1.000	0.01699	−0.63631	1.73577	−2.182
Vietnam	8	8	1.000	0.01970	−0.68169	1.66949	−2.560
Malaysia	18	18	1.000	0.01589	−1.00096	1.22653	−13.471**
Indonesia	4	4	1.000	0.01682	0.10809	–	−0.065
Taiwan	9	9	1.000	0.01395	−0.50053	–	−4.294**
Benin	10	9	0.978	0.02895	−0.27603	−0.39901	−1.149
Kenya	10	9	0.978	0.02254	−0.45487	1.67459	−1.727
Papua New Guinea (including Fiji)	19	18	0.994	0.01760	−0.50193	–	−10.644**
Colombia	4	2	0.667	0.02191	2.2542	–	5.480
All countries	105	92	0.997	0.02391	−1.43781	1.07414	−33.432**
*MvitPBP2*							
India (including Bangladesh)	5	5	1.000	0.01657	0.03603	−7.12783	−0.608
Thailand	4	4	1.000	0.01044	−0.52807	1.24589	−0.480
Cambodia	6	3	0.800	0.01616	1.34234	0.63605	4.187
Laos	6	3	0.800	0.01293	1.32483	0.64579	3.583
Vietnam	6	6	1.000	0.01455	−0.20433	0.22587	−1.489
Malaysia	9	8	0.972	0.01111	−1.04552	1.09656	−2.459
Indonesia	4	2	0.667	0.00808	2.15629	–	3.526
Taiwan	8	8	1.000	0.01371	−0.62621	3.53310	−3.074*
Benin	18	18	1.000	0.01406	−1.63769	1.30841	−13.228**
Kenya	15	9	0.914	0.01308	−0.17671	−62.00550	−0.342
Papua New Guinea (including Fiji)	13	13	1.000	0.01608	−1.10752	1.70594	−6.801**
Colombia	4	1	0.000	0.00000	–	–	–
All countries	98	77	0.994	0.02507	−1.02576	0.66958	−32.919**
*MvitPBP3*							
India (including Bangladesh)	5	5	1.000	0.01546	−0.41429	2.25089	−0.696
Thailand	4	4	1.000	0.01305	−0.84307	0.72862	−0.187
Laos	5	5	1.000	0.01948	−0.60389	1.42504	−0.379
Vietnam	4	3	0.833	0.01841	0.51295	4.12994	2.479
Malaysia	6	6	1.000	0.01740	−0.63026	2.51480	−1.181
Indonesia	4	4	1.000	0.01707	−0.85057	0.83784	0.142
Taiwan	5	5	1.000	0.01084	−0.45202	3.26180	−1.223
Benin	6	6	1.000	0.01794	−1.35908	1.15655	−1.133
Kenya	11	8	0.891	0.01380	−0.89451	−0.03719	−0.642
Papua New Guinea (including Fiji)	13	13	1.000	0.01550	−1.30434	1.17621	−6.957**
Colombia	5	5	1.000	0.02771	−0.54307	1.96967	0.075
All countries	68	64	0.997	0.02501	−1.73328	1.13753	−33.411**

* Values were significant at *p* < .01.

** Values were significant at *p* < .001.

When the *Maruca* samples were analyzed by continent, the highest nucleotide diversity based on *MvitPBP1* was recorded for *M. vitrata* populations from Africa (0.02544), followed by South America (Table 4). The nucleotide diversity was almost similar for both Asia and Oceania *Maruca* populations. Nucleotide diversity based on *MvitPBP2* was almost similar for all continents, except South America, which was the lowest (Table 4). However, it was the highest based on *MvitPBP3* for South America and it was almost similar for all other continents (Table 4). On continental basis as well, the haplotype diversity was one or close to one for most of the sampled continents for all the *MvitPBP* genes (Table 4). The lowest haplotype diversity was recorded for South America, only based on *MvitPBP1* and *MvitPBP2* genes, since we used only the Colombia samples to represent South America.

**Table 4:** List of number of samples studied, number of haplotypes, haplotype diversity (*h*), nucleotide diversity (π), Tajima's *D* and Fu's *F_S_* tests for *Maruca* spp. populations from four selected continents

Continent	No. of samples	No. of haplotypes	Haplotype diversity (*h*)	Nucleotide diversity (π)	Tajima's *D*	Tajima's D (NonSyn/Syn) ratio	Fu's *F_S_*
*MvitPBP1*							
Africa	20	18	0.989	0.02544	−0.65850	1.01195	−6.633**
Asia	62	54	0.995	0.01730	−1.07993	1.11037	−33.341**
Oceania	19	18	0.994	0.01760	−0.50193	–	−10.644**
South America	4	2	0.667	0.02191	2.2542	–	5.480
*MvitPBP2*							
Africa	33	27	0.983	0.01383	−1.58695	1.26913	−17.355**
Asia	48	38	0.991	0.01493	−1.04512	1.23461	−28.646**
Oceania	13	13	1.000	0.01608	−1.10752	1.70594	−6.801**
South America	4	1	0.000	0.00000	–	–	–
*MvitPBP3*							
Africa	17	14	0.956	0.01536	−1.39236	0.47638	−4.452**
Asia	33	32	0.998	0.01725	−1.43319	2.06989	−27.663**
Oceania	13	13	1.000	0.01550	−1.30434	1.17621	−6.957**
South America	5	5	1.000	0.02771	−0.54307	1.96967	0.075

* Values were significant at *p* < .01.

** Values were significant at *p* < .001.

Based on *MvitPBP1*, Tajima's *D* value was positive only for India, Cambodia, Indonesia, and Thailand, with Colombia being the highest (2.2542) (Table 3). Based on *MvitPBP2*, Tajima's *D* value was positive only for India, Cambodia, Indonesia, and Laos (Table 3). Tajima's *D* value for *MvitPBP3* was negative and nonsignificant, except for Vietnam populations. On continental basis as well, the Tajima's *D* value was negative for most of the sampled continents for all the *MvitPBP* genes, except South America for *MvitPBP1* and *MvitPBP2* genes, but Colombia was the only representative of South America (Table 4).

Apart from the India, Thailand, Cambodia, and Colombia *Maruca* samples, all other populations showed negative values for Fu's *F_S_* test with or without significance based on *MvitPBP1* (Table 3). *Maruca* populations from Cambodia, Indonesia, and Laos showed positive Fu's *F_S_* values without significance for *MvitPBP2* (Table 3). Similarly, *Maruca* populations from Colombia, Indonesia, and Vietnam showed positive Fu's *F_S_* values without significance for *MvitPBP3* (Table 3). The total Fu's *F_S_* values of all *Maruca* populations were negative and highly significant for all the three genes. On continental basis, the results were similar to Tajima's *D* test. All the Fu's *F_S_* values were negative for the sampled continents for all the *MvitPBP* genes, except South America, which was represented only by Colombia (Table 4).

### 3.4 *F*‐statistics (*F*_ST_) and analysis of molecular variance

The *F*_ST_ values of all pairwise comparisons for *MvitPBP1*, *MvitPBP2,* and *MvitPBP3* ranged from −0.0084 to 0.7405, −0.0911 to 0.8273, and −0.0089 to 0.6900, respectively (Tables 5, 6, 7). Negative *F*_ST_ values indicate the absence of genetic differences between the two compared populations (Jaramillo, Montaña, Castro, Vallejo, & Guhl, 2001). Based on the negative *F*_ST_ values obtained for *MvitPBP1*, *Maruca* populations from Asia and Oceania were similar to each other, and the *M. vitrata* populations from Kenya and Benin were similar to each other (Table 5). Among the Asia and Oceania *Maruca* populations, India, Taiwan, Thailand, Vietnam, and PNG populations were similar based on pairwise *F*_ST_ values obtained for *MvitPBP2* (Table 6). India, Indonesia, Laos, Malaysia, and PNG populations were similar based on pairwise *F*_ST_ values obtained for *MvitPBP3* (Table 7). Significant differences (*F*_ST_: 0.5438–0.7405, *p* < .05) were obtained for Colombian *Maruca* populations with all other populations, as well as African *M. vitrata* populations from all other populations (*F*_ST_: 0.1936–0.6062, mostly *p* < .01). The genetic difference of Colombia *Maruca* populations from all other populations based on *MvitPBP2* (*F*_ST_: 0.4472–0.8273, *p* < .05) (Table 6) and *MvitPBP3* (0.5712–0.6900; *p* < .05) was significant (Table 7). Similarly, the genetic difference of both the Benin and Kenya populations from all other *Maruca* populations for *MvitPBP2* (0.6088–0.7260; *p* < .01) and from all other *Maruca* populations except Vietnam for *MvitPBP3* (0.2317–0.6900; *p* < .01) was highly significant.

**Table 5:** Pairwise *F*_ST_ values (below diagonal) and the statistical significance (above diagonal) comparing populations of *Maruca* spp. based on PBP1

Population	1	2	3	4	5	6	7	8	9	10	11	12
1. Colombia	.0000	**	**	*	**	*	**	**	**	*	**	**
2. Papua New Guinea	.6817	.0000	ns	ns	*	**	ns	ns	*	ns	**	**
3. Malaysia	.6981	.0024	.0000	ns	*	**	ns	ns	ns	ns	**	**
4. Indonesia	.6790	−.0705	−.0335	.0000	ns	*	ns	ns	ns	ns	**	**
5. Laos	.6609	.0630	.0924	.0967	.0000	*	*	ns	ns	ns	**	**
6. Cambodia	.6358	.2305	.2775	.2871	.1624	.0000	ns	*	**	*	**	**
7. Thailand	.7405	.0112	.0403	−.0330	.1881	.3423	.0000	ns	ns	ns	**	**
8. Vietnam	.6441	−.0084	.0037	−.0477	−.0120	.2221	.0280	.0000	ns	ns	**	**
9. Taiwan	.7129	.0477	.0366	−.0350	.0907	.3415	.0578	−.0326	.0000	ns	**	**
10. India	.6673	−.0451	.0091	−.1406	−.0300	.2339	.0056	−.1043	−.0868	.0000	**	*
11. Benin	.5438	.2718	.2598	.1943	.2220	.3282	.2746	.1966	.2310	.1936	.0000	ns
12. Kenya	.6062	.2968	.2908	.2419	.2760	.4053	.3111	.2312	.2738	.2357	−.0238	.0000

Abbreviation: ns, nonsignificant.

* *F*_ST_ values were significant at *p* < .05.

** Highly significant at *p* < .01.

**Table 6:** Pairwise *F*_ST_ values (below diagonal) and the statistical significance (above diagonal) comparing populations of *Maruca* spp. based on PBP2

Population	1	2	3	4	5	6	7	8	9	10	11	12
1. Colombia	.0000	**	**	*	**	*	**	**	**	**	**	**
2. Papua New Guinea	.4472	.0000	*	**	*	ns	**	ns	ns	ns	**	**
3. Malaysia	.6572	.0558	.0000	**	**	ns	*	ns	**	**	**	**
4. Indonesia	.8273	.1851	.4004	.0000	**	*	**	*	**	**	**	**
5. Cambodia	.5700	.0795	.1980	.2765	.0000	ns	**	ns	*	ns	**	**
6. Thailand	.7357	−.0911	.0203	.3480	.0909	.0000	ns	ns	ns	ns	**	**
7. Laos	.6631	.0986	.1273	.3301	.2103	.1104	.0000	ns	ns	*	**	**
8. Vietnam	.5875	−.0384	.0570	.2594	.0216	−.0758	.0867	.0000	ns	ns	**	**
9. Taiwan	.5695	−.0183	.1375	.3014	.1037	−.0688	.0892	.0368	.0000	*	**	**
10. India	.5473	.0166	.1402	.2733	.0034	.0250	.1890	−.0111	.0923	.0000	**	**
11. Benin	.7062	.6089	.6410	.6889	.6315	.6244	.6411	.6252	.6277	.6088	.0000	ns
12. Kenya	.7260	.6131	.6518	.7038	.6406	.6413	.6502	.6345	.6394	.6156	.0295	.0000

Abbreviation: ns, nonsignificant.

* *F*_ST_ values were significant at *p* < .05.

** Highly significant at *p* < .01.

**Table 7:** Pairwise *F*_ST_ values (below diagonal) and the statistical significance (above diagonal) comparing populations of *Maruca* spp. based on PBP3

Population	1	2	3	4	5	6	7	8	9	10	11
1. Colombia	.0000	**	**	*	**	*	*	*	**	**	**
2. Papua New Guinea	.6626	.0000	ns	ns	*	*	**	ns	ns	**	**
3. Malaysia	.6073	−.0089	.0000	ns	ns	ns	ns	ns	*	**	**
4. Indonesia	.6001	−.0072	−.0361	.0000	*	ns	*	ns	**	**	**
5. Laos	.5998	.0924	.0587	.0907	.0000	ns	ns	*	**	**	**
6. Vietnam	.5712	.1473	.1221	.0882	.0002	.0000	ns	ns	**	*	*
7. India	.6122	.0753	.0388	.1029	−.0407	.0733	.0000	ns	*	**	**
8. Thailand	.6341	.0038	.0336	.0164	.1080	.1966	.1344	.0000	ns	**	**
9. Taiwan	.6671	.0623	.0696	.0989	.1221	.2449	.1516	.1108	.0000	**	**
10. Benin	.6245	.3618	.3053	.2979	.2317	.0963	.2609	.4194	.4378	.0000	ns
11. Kenya	.6900	.4094	.3875	.3830	.3162	.1594	.3405	.4834	.4935	.0233	.0000

Abbreviation: ns, nonsignificant.

* *F*_ST_ values were significant at *p* < .05.

** Highly significant at *p* < .01.

Based on continental analysis, the *F*_ST_ values of all population pairwise comparisons for *MvitPBP1*, *MvitPBP2,* and *MvitPBP3* ranged from −0.0968 to 0.6840, −0.0073 to 0.7260, and −0.0042 to 0.6900, respectively (Tables 8, 9, 10). Based on the negative *F*_ST_ values obtained for *MvitPBP1*, South Asia and Oceania (PNG), Oceania (Fiji) and Southeast Asia, and East and West Africa *Maruca* populations were similar (Table 8). The genetic difference of South America *Maruca* populations from all other populations was significant (*F*_ST_: 0.5438–0.6840; *p* < .05). Similarly, the genetic difference of Africa *M. vitrata* populations from all other populations was significant. However, West Africa *M. vitrata* populations did not differ significantly from Oceania (Fiji) (*F*_ST_ = 0.1223) (Table 8). Interestingly, Oceania (Fiji) *Maruca* population was not significantly different from South and Southeast Asia as well as South America populations based on pairwise *F*_ST_ values obtained for *MvitPBP2* (Table 9). The genetic difference of Africa *M. vitrata* populations was highly significant with other regions (0.6088–0.7260; *p* < .01). Based on the *F*_ST_ values obtained for *MvitPBP3*, Oceania and Southeast Asia *Maruca* populations were similar (Table 10). However, the difference between South America (0.5000–0.6900; *p* < .01) or Africa (0.2609–0.6900; *p* < .01) *Maruca* populations and all other populations except Oceania (Fiji) was highly significant.

**Table 8:** Pairwise *F*_ST_ values (below diagonal) and the statistical significance (above diagonal) comparing populations of *Maruca vitrata* PBP1

Populations	1	2	3	4	5	6	7
1. South America (Colombia)	.0000	**	ns	**	*	**	**
2. Oceania (PNG)	.6840	.0000	ns	ns	ns	**	**
3. Oceania (Fiji)	.6168	.0929	.0000	ns	ns	ns	*
4. Asia (Southeast)	.6821	.0060	.0488	.0000	ns	**	**
5. Asia (South)	.6673	−.0366	−.0968	−.0523	.0000	*	**
6. Africa (West)	.5438	.2706	.1223	.2805	.1936	.0000	ns
7. Africa (East)	.6062	.2959	.2660	.2941	.2357	−.0238	.0000

Abbreviation: ns, nonsignificant.

* *F*_ST_ values were significant at *p* < .05.

** Highly significant at *p* < .01.

**Table 9:** Pairwise *F*_ST_ values (below diagonal) and the statistical significance (above diagonal) comparing populations of *Maruca vitrata* PBP2

Populations	1	2	3	4	5	6	7
1. South America (Colombia)	.0000	**	ns	**	**	**	**
2. Oceania (PNG)	.5482	.0000	*	ns	ns	**	**
3. Oceania (Fiji)	.4133	.1184	.0000	ns	ns	**	**
4. Asia (Southeast)	.4807	−.0073	.1049	.0000	ns	**	**
5. Asia (South)	.5473	.0310	.0676	.0529	.0000	**	**
6. Africa (West)	.7062	.6255	.6119	.6183	.6088	.0000	ns
7. Africa (East)	.7260	.6316	.6261	.6194	.6156	.0295	.0000

Abbreviation: ns, nonsignificant.

* *F*_ST_ values were significant at *p* < .05.

** Highly significant at *p* < .01.

**Table 10:** Pairwise *F*_ST_ values (below diagonal) and the statistical significance (above diagonal) comparing populations of *Maruca vitrata* PBP3

Populations	1	2	3	4	5	6	7
1. South America (Colombia)	.0000	**	ns	**	**	**	**
2. Oceania (PNG)	.6555	.0000	ns	ns	ns	**	**
3. Oceania (Fiji)	.5000	−.2215	.0000	ns	ns	ns	ns
4. Asia (Southeast)	.6552	−.0042	−.0980	.0000	ns	**	**
5. Asia (South)	.6122	.0651	.0128	.0220	.0000	**	**
6. Africa (West)	.6245	.3499	.3128	.2904	.2609	.0000	ns
7. Africa (East)	.6900	.4014	.4400	.3320	.3405	.0233	.0000

Abbreviation: ns, nonsignificant.

* *F*_ST_ values were significant at *p* < .05.

** Highly significant at *p* < .01.

AMOVA analysis showed that there is relatively little differentiation among populations within the same region/continent for *MvitPBP1* (Φ_SC_ = −0.0157), *MvitPBP2* (Φ_SC_ = −0.0575) and *MvitPBP3* (Φ_SC_ = −0.0078) (Tables 11, 12, 13). Both the differences between populations of different regions/continents (Φ_CT_ = 0.3191, 0.5342 and 0.4116 for *MvitPBP1*, *MvitPBP2* and *MvitPBP3*, respectively) and the differences within all populations in various region/continent (Φ_ST_ = 0.3084, 0.5610 and 0.4070 for *MvitPBP1*, *MvitPBP2* and *MvitPBP3*, respectively) are almost equally responsible for all of the differences. Thus, most of the genetic variation occurred within populations (43.90%–69.16%) as well as among the regions/continents (31.91%–53.42%), with much smaller amounts occurring among populations.

**Table 11:** Result of AMOVA analysis of *Maruca* spp. populations from four selected continents/ regions based on PBP1 sequence data

Source of variation	*df*	Sum of squares	Variance components	Percentage of variation	Fixation indices
Among continents/ regions	3	125.83	1.91*	31.91	Φ_CT_ = 0.3191
Among populations within continents/ regions	3	11.18	−0.06^ns^	−1.07	Φ_SC_ = −0.0157
Within all populations	98	405.73	4.14**	69.16	Φ_ST_ = 0.3084
Total	104	542.74	5.99		

* Significant at *p* < .05.

** Highly significant at *p* < .01.

**Table 12:** Result of AMOVA analysis of *Maruca* spp. populations from four selected continents/ regions based on PBP2 sequence data

Source of variation	*df*	Sum of squares	Variance components	Percentage of variation	Fixation indices
Among continents/ regions	3	281.33	4.26*	53.42	Φ_CT_ = 0.5342
Among populations within continents/ regions	3	16.89	0.21**	2.68	Φ_SC_ = −0.0575
Within all populations	91	318.49	3.50**	43.90	Φ_ST_ = 0.5610
Total	97	616.70	7.97		

* Significant at *p* < .05.

** Highly significant at *p* < .01.

**Table 13:** Result of AMOVA analysis of *Maruca* spp. populations from four selected continents/ regions based on PBP3 sequence data

Source of variation	*df*	Sum of squares	Variance components	Percentage of variation	Fixation indices
Among continents/ regions	3	144.67	2.96*	41.16	Φ_CT_ = 0.4116
Among populations within continents/ regions	3	12.21	−0.03^ns^	−0.46	Φ_SC_ = −0.0078
Within all populations	61	260.34	4.27**	59.30	Φ_ST_ = 0.4070
Total	67	417.24	7.20		

* Significant at *p* < .05.

** Highly significant at *p* < .01.

### 3.5 Phylogenetic pattern based on *MvitPBPs*

**Figure 3:**
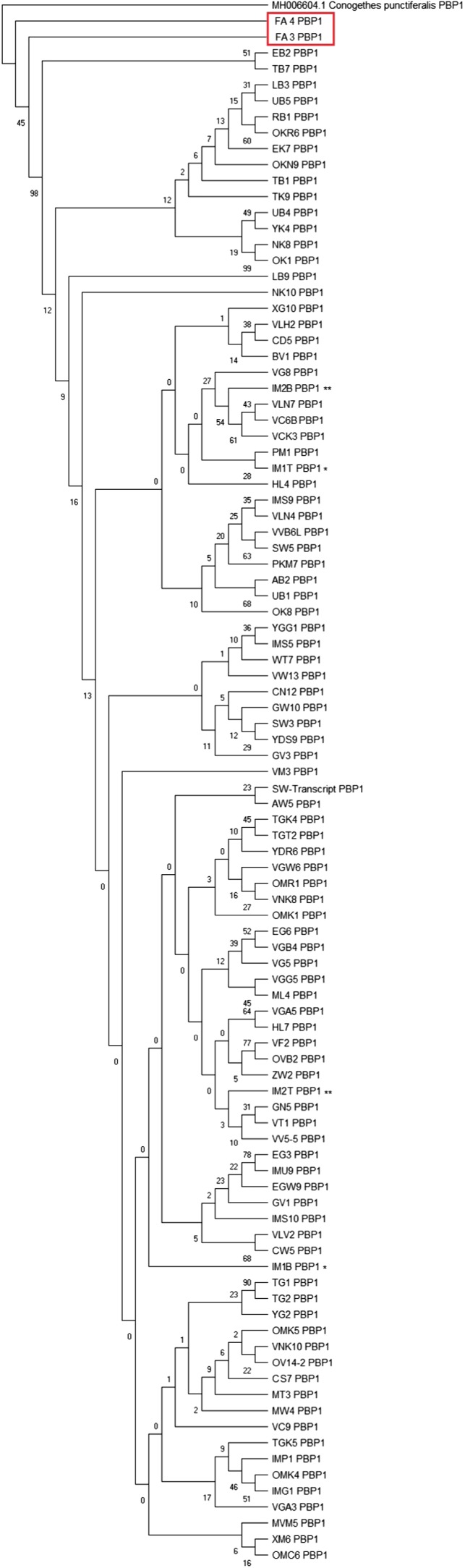
Phylogenetic relationship among *Maruca* sp. based upon a 426 bp *MvitPBP1* gene fragments using maximum likelihood (ML) algorithm. The South American *Maruca* group is marked in red. Isoforms of *MvitPBP1* gene in selected individuals of different populations are shown with asterisk mark. Refer to Appendix 1 for the *Maruca* population details used in this study

**Figure 4:**
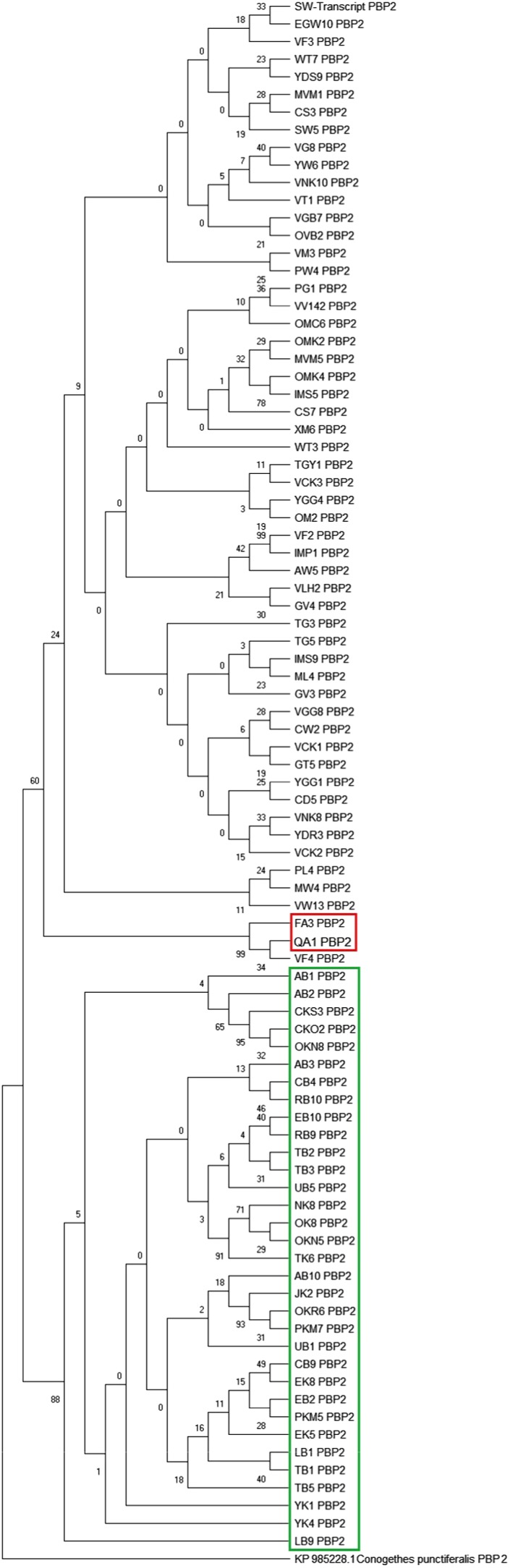
Phylogenetic relationship among *Maruca* sp. based upon a 495 bp *MvitPBP2* gene fragments using maximum likelihood (ML) algorithm. The South American *Maruca* group is marked in red, whereas African *Maruca* group is marked in green. Refer to Appendix 2 for the *Maruca* population details used in this study

**Figure 5:**
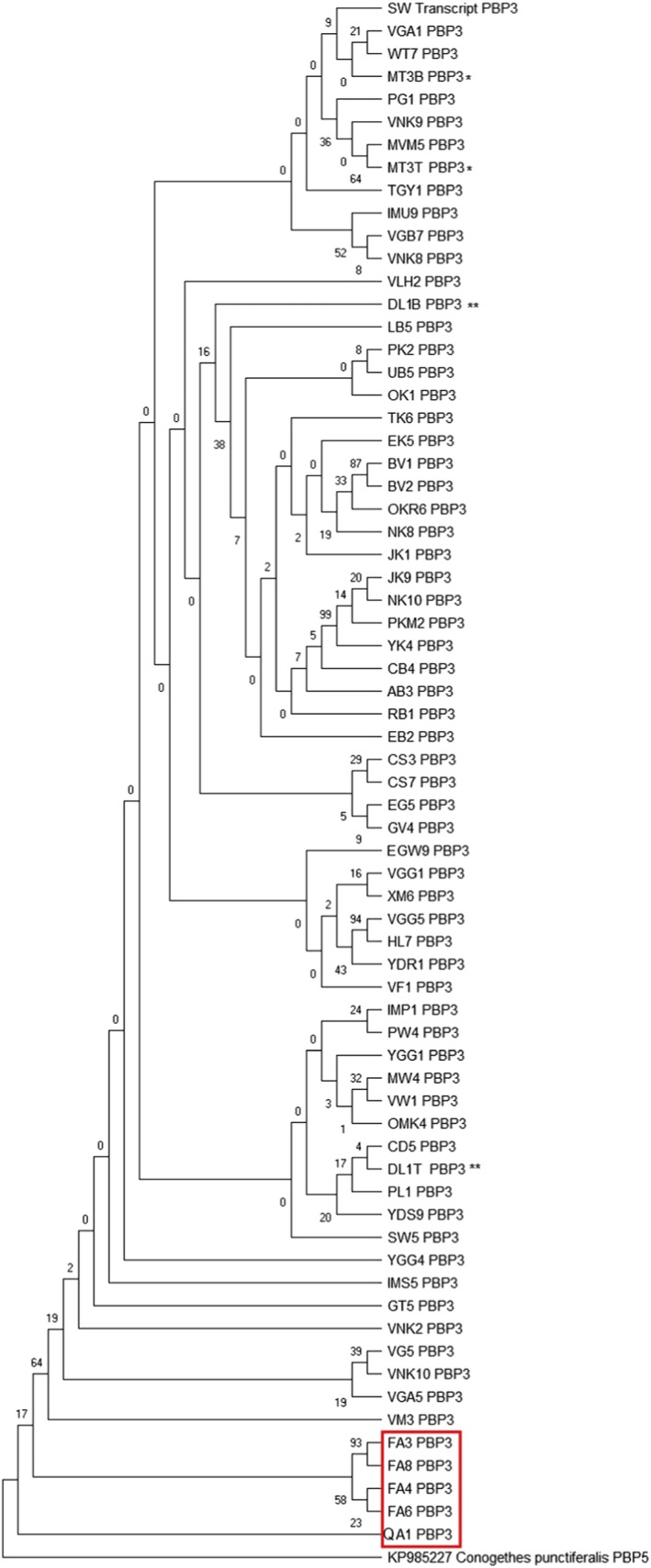
Phylogenetic relationship among *Maruca* sp. based upon a 498 bp *MvitPBP3* gene fragments using maximum likelihood (ML) algorithm. The South American *Maruca* group is marked in red. Isoforms of *MvitPBP3* gene in selected individuals of different populations are shown with asterisk mark. Refer to Appendix 3 for the *Maruca* population details used in this study

The intraspecific phylogenetic relationships of *MvitPBP1* cDNA among *Maruca* populations from Asia, Africa, Oceania, and South America are shown in Figure 3. All *Maruca* populations formed a single cluster, except South America (Colombia), which formed a separate clade. However, intraspecific phylogenetic relationships based on *MvitPBP2* cDNA among *Maruca* populations from target continents formed a separate clade for the *M. vitrata* populations from Africa (88% bootstrap support, Figure 4). Interestingly, the South American populations aligned within the Asia/Oceania clade, although it formed a separate subclade with 99% bootstrap value. One of the samples from South America (QA1) fully aligned with an Oceania (Fiji) sample (VF4). The intraspecific phylogenetic relationships of *MvitPBP3* cDNA were similar to MvitPBP1, with one clade for all samples except those from Colombia (Figure 5).

### 3.6 Automatic barcoding gap discovery

**Figure 6:**
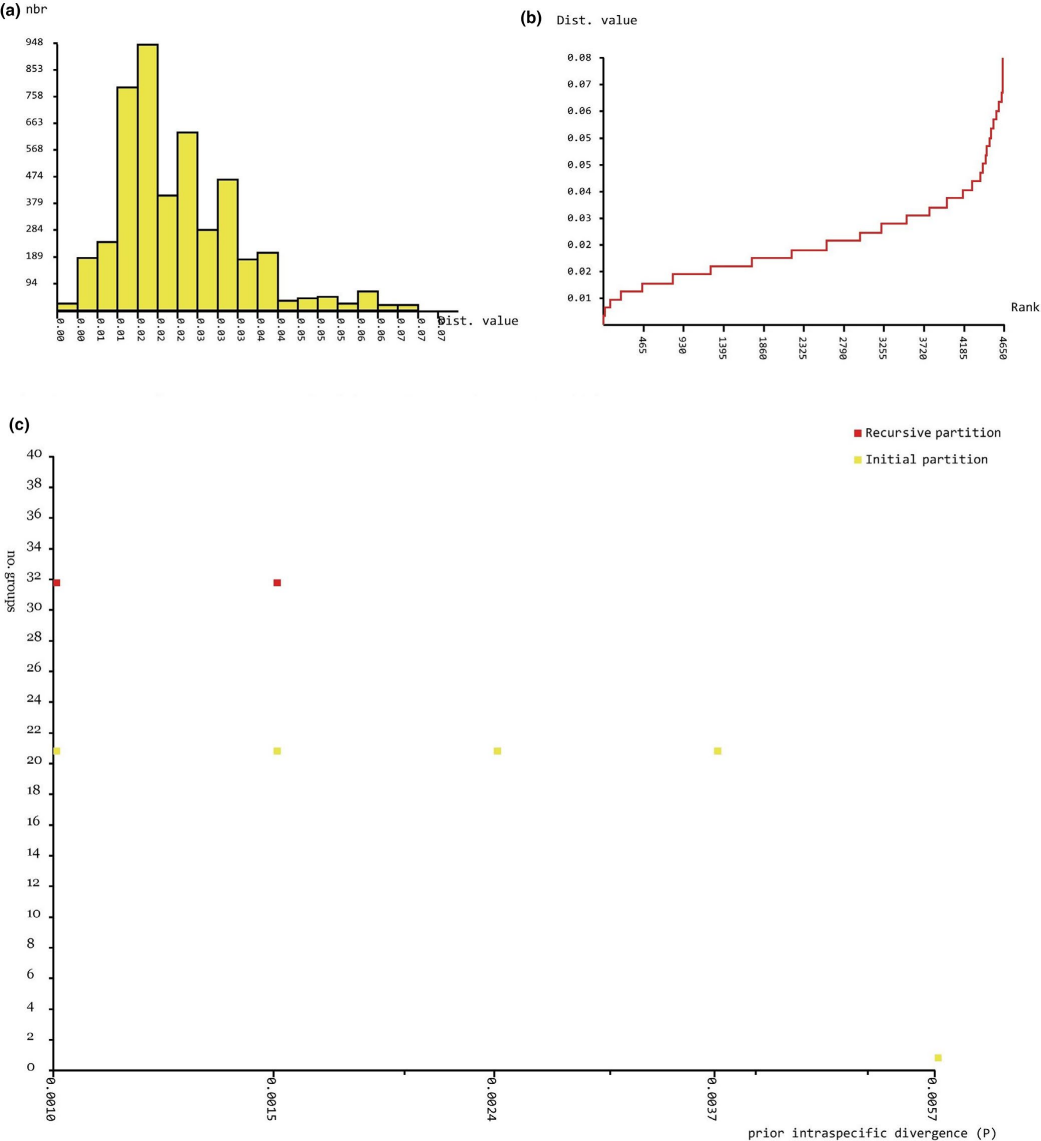
ABGD analysis based on *MvitPBP1*—Distribution of *Maruca* spp. population K2P mean divergence in (a) histogram of distances, (b) ranked distances, and (c) ABGD partition

**Figure 7:**
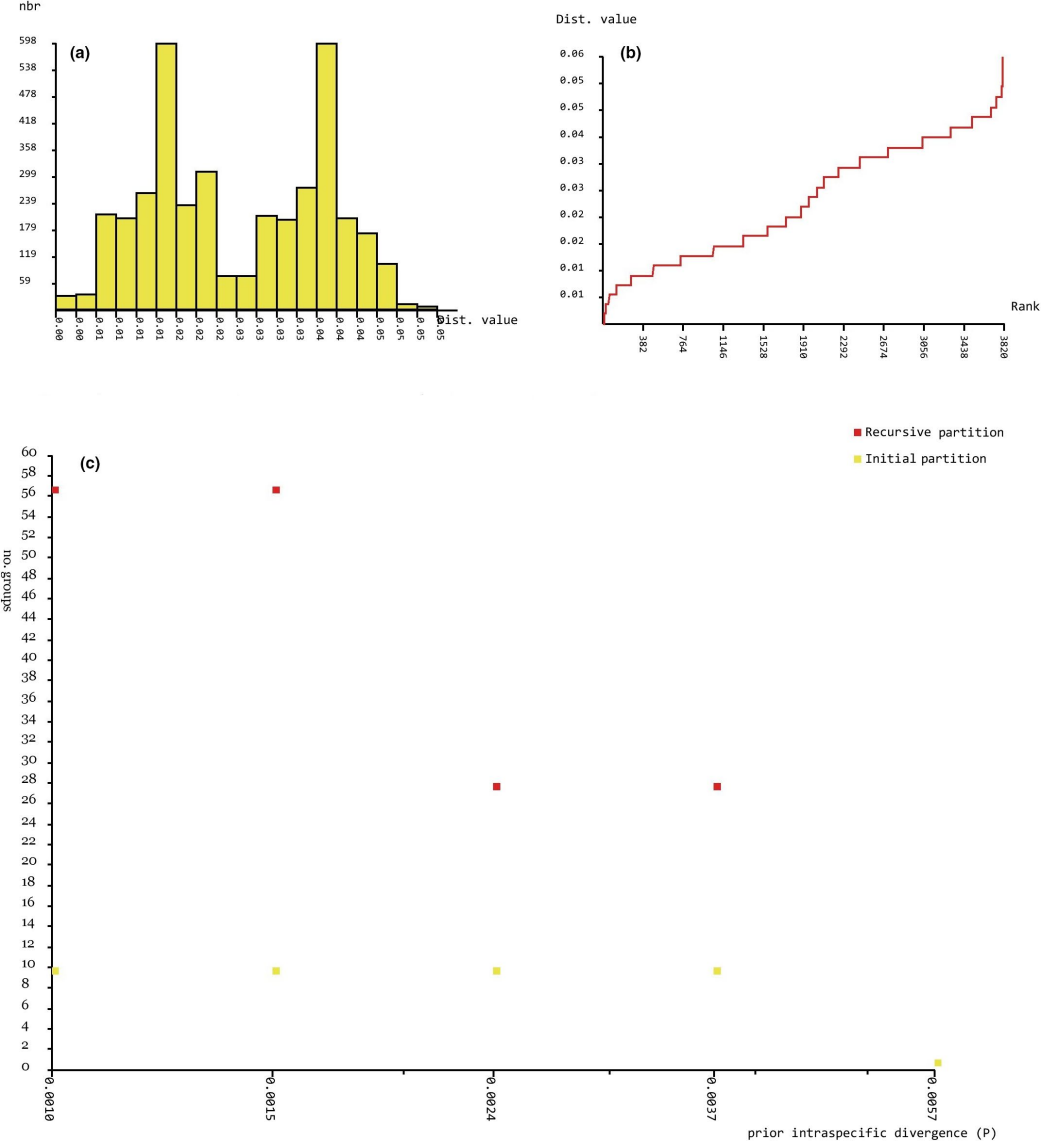
ABGD analysis based on *MvitPBP2*—Distribution of *Maruca* spp. population K2P mean divergence in (a) histogram of distances, (b) ranked distances, and (c) ABGD partition

**Figure 8:**
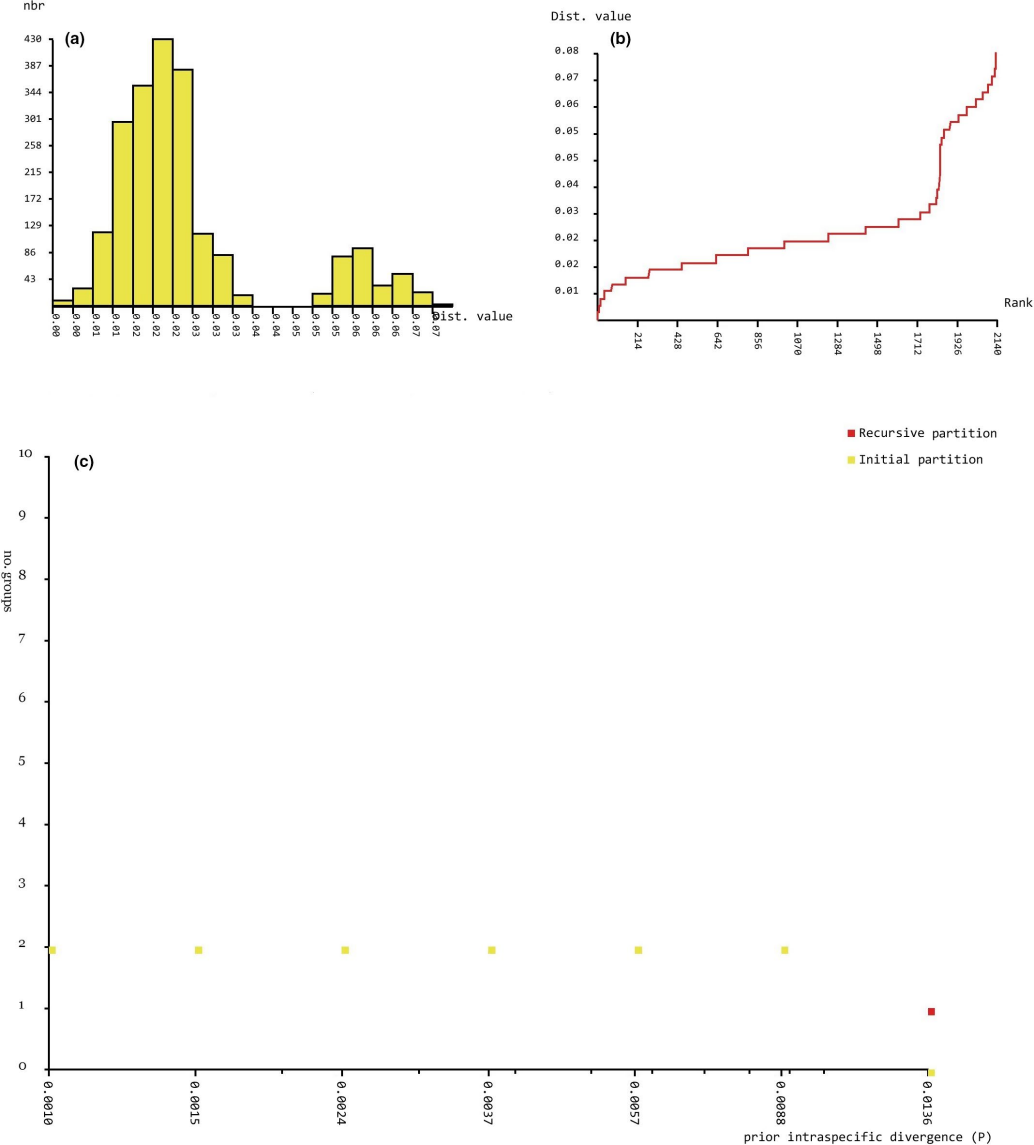
ABGD analysis based on *MvitPBP3—*Distribution of *Maruca* spp. population K2P mean divergence in (a) histogram of distances, (b) ranked distances, and (c) ABGD partition

ABGD analysis of *MvitPBP1* resulted in four partitions with a prior of intraspecific divergence up to 0.004 (Figure 6a–c). The results showed the existence of 21 groups among the study populations (Table 14). Although few populations from Cambodia, Laos, Malaysia, Taiwan, Benin, and Kenya formed separate groups, the major group contained most of the *Maruca* populations from Asia, Africa, and Oceania. The only clear separation without overlapping was the *Maruca* populations from South America, and thus the ABGD result is congruent with the phylogenetic tree based on *MvitPBP1*. ABGD analysis of *MvitPBP2* also resulted in four partitions with a prior of intraspecific divergence up to 0.004 (Figure 7a–c). The analysis suggested the presence of 10 groups (Table 15), confirming the phylogenetic results for *MvitPBP2*. For instance, the *Maruca* populations from Africa formed a separate group from another major group containing Asia and PNG populations. The South America populations formed a separate group and one of the Oceania (Fiji) samples also aligned with this group. ABGD analysis of *MvitPBP3* resulted in six partitions with a prior of intraspecific divergence up to 0.009 (Figure 8a–c). There were only two groups for *MvitPBP3* (Table 16). As showed in the phylogenetic tree, ABGD analysis for *MvitPBP3* also suggests a single group for *Maruca* populations from Asia, Africa, and Oceania, whereas South America populations formed a separate group.

**Table 14:** List of identified *Maruca vitrata* PBP1 groups based on ABGD analysis

Group	Population	Frequency
1	IMP1, OMK1, OMK4, OMK5, OMR1, PM1, VM3, IMG1, IMS5, IMS9, IMS10, XM6, OMC6, IM1L, IM1U, IM2U, IMU9, VNK8, VNK10, CN12, GN5, HL4, HL7, ML4, VLH2, VLN4, VLV2, VC9, MT3, VT1, WT7, BV1, GV1, GV3, MVM5, OV14−2, OVB2, VV5−5, VVB6L, AW5, CW5, GW10, MW4, SW5, VW13, ZW2, CD5, YDR6, YDS9, CG7, SW1, AB2, UB1, OK8, PKM7, XG10, TG1, TG2, TGK4, TGK5, TGT2, UG3, UG6, UGW9, VG5, VG8, VGA3, VGA5, VGB4, VGG5, VGW6, YG2, YGG1, VF2	74
2	IM2L	1
3	VLN7, VCK3	2
4	VC6L	1
5	SW3	1
6	EB2	1
7	LB3	1
8	LB9	1
9	RB1, OKR6	2
10	TB7	1
11	TB1	1
12	UB4	1
13	UB5	1
14	EK7	1
15	NK8, OK1	2
16	NK10	1
17	OKN9	1
18	TK9	1
19	YK4	1
20	FA3	1
21	FA4	1

**Table 15:** List of identified *Maruca vitrata* PBP2 groups based on ABGD analysis

Group	Population	Frequency
1	SW1, PG1, TGY1, TG3, TG5, EGW10, VG8, VGB7, VGG8, YGG1, YGG4, VF3, OMK2, OMK4, VM3, OM2, OMC6, XM6, IMS5, IMS9, VNK8, VNK10, VCK1, VCK2, VCK3, GT5, VT1, WT3, WT7, ML4, PL4, VLH2, GV3, MVM1, MVM5, OVB2, VV142, CW2, MW4, PW4, VW13, YW6, CS3, CS7, CD5, YDR3, YDS9	47
2	FA3, FA1‐T, VF4	3
3	VF2, IMP1	2
4	GV4	1
5	AW5	1
6	SW5	1
7	AB1, AB2, AB3, CB4, CB9, EB2, EB10, LB1, LB9, RB9, RB10, TB1, TB3, UB1, UB5, CKS3, CKO2, EK5, EK8, JK2, NK8, OK8, OKN5, OKN8, OKR6, PKM5, PKM7, TK6, YK1, YK4	30
8	AB10	1
9	TB2	2
10	TB5	1

**Table 16:** List of identified *Maruca vitrata* PBP3 groups based on ABGD analysis

Group	Population	Frequency
1	SW1, PG1, TGY1, UG5, UGW9, VG5, VGG1, VGG5, VGB7, VGA1, VGA5, YGG1, YGG4, VF1, IMP1, OMK4, VM3, IMS5, IMU9, XM6, VNK2, VNK8, VNK9, VNK10, DL1, HL7, PL1, VLH2, BV1, BV2, GV4, MVM5, CG3, CG7, CD5, YDR1, YDS9, GT5, MT3, WT7, MW4, PW4, SW5, VW1, AB3, CB4, EB2, LB5, UB5, RB1, EK5, JK1, JK9, NK8, NK10, OK1, OKR6, PK2, PKM2, TK6, YK4	61
2	FA3, FA4, FA6, FA8, TA1	5

### 3.7 Haplotype network

**Figure 9:**
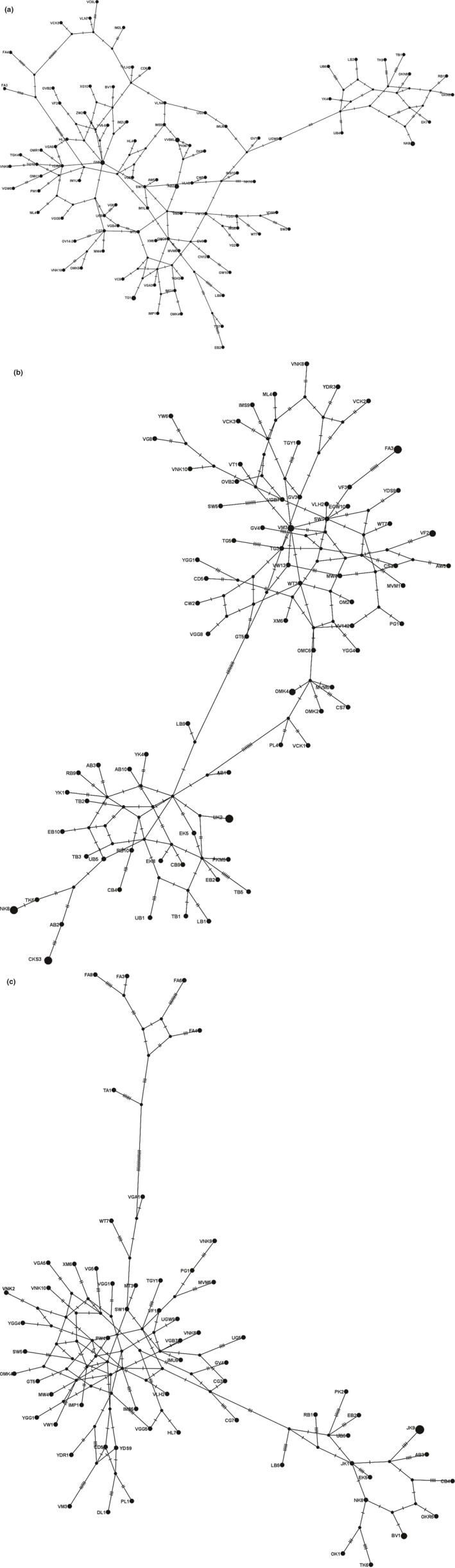
Haplotype network of the *MvitPBP* genes of *Maruca* spp. in Asia, Africa, Oceania, and South America. (a) *MvitPBP1* haplotypes, (b) *MvitPBP2* haplotypes, and (c) *MvitPBP3* haplotypes found in the study were included in the network analyses. Haplotype frequency is represented by the size of each node

The haplotype network analysis involving the active *MvitPBP1* haplotypes in this study revealed two distinct groups (Figure 9a). Although the phylogenetic tree and the ABGD grouping clearly differentiated the South America *Maruca* populations from rest of the populations, they were placed at the periphery of the radial expansion of the major cluster that contained the Asian and Oceania populations in the haplotype network. Surprisingly, few African populations also aligned within this cluster. However, majority of the African populations formed a separate cluster. Similar clustering was also obtained for the network based on active *MvitPBP2* haplotypes (Figure 9b). However, the results from the network based on active *MvitPBP3* haplotypes clearly differentiated the populations in this study into three clusters—Asia and Oceania as the major cluster, Africa and South America as the two other minor, but distinct clusters (Figure 9c).

## 4 DISCUSSION

The PBPs were not studied in detail in *M. vitrata*, until our preliminary first report (Malini, Schafleitner, Muthukalingan, & Srinivasan, 2013), where we reported the phylogeny of *M. vitrata* based on *MvitPBP* sequences. Subsequently, the role of PBPs in sex pheromone perception in *M. vitrata* was studied in China (Mao et al., 2016), but PBPs were never used in population genetics of this organism. Since geographically distinct *Maruca* populations were identified using *coxI* (Malini et al., 2015) and ITS2 (Malini, Schafleitner, Srinivasan, & Krishnan, 2014), the differences in the protein coding sequences of *Maruca* PBPs from Asia, Africa, Oceania, and South America were characterized in this study. Identification of variation in PBP proteins is likely to provide insights on differences in pheromone response of *Maruca* populations.

Two different PBPs were identified for the first time from male moths of *M. vitrata* and named as MvitPBP1 and MvitPBP2. Although they were published in NCBI (KF006811.1–KF006814.1), Mao et al. (2016) did not include them in their phylogenetic analysis. Another PBP was identified from *M. vitrata* female adults and named as MvitPBP3. It is common to have more than one PBP in moth species. Earlier studies also reported the occurrence of multiple PBPs in moths that produce multi‐component sex pheromones, and each PBP may be encoded by a distinct locus (Newcomb, Sirey, Rassam, & Greenwood, 2002). Hence, it is possible that each PBP recognizes a specific compound in the multi‐component pheromone blend. For instance, two PBPs were described in *Lymantria dispar* (Vogt, Köhne, Dubnau, & Prestwich, 1989), which selectively bound the two pheromone enantiomers (Bette et al., 2002; Du & Prestwich, 1995; Plettner, Lazar, Prestwich, & Prestwich, 2000). Although one of the three PBPs from *Antheraea polyphemus* (ApolPBP1) was shown to bind to all three pheromone compounds with high affinity at high pH, competitive assays showed considerable differences in affinity only for the major compound (Leal, Chen, & Erickson, 2005). Thus, the occurrence of three PBPs in *M. vitrata* moths could be related to the three component nature of its sex pheromone blend.

The structure of the PBP gene sequences and proteins was well described in *B. mori* and *A. polyphemus* (Sandler et al., 2000; Yu et al., 2012). Usually, the PBP genes encode peptides of about 140 amino acids. All the three MvitPBPs, including signal peptides, are composed of slightly over 150 amino acids with six cysteine residues. Sequences of Lepidopteran PBPs showed a conserved motif of six cysteine residues linked by three disulfide bonds to provide a hydrophobic pocket (Breer, Krieger, & Raming, 1990). In addition, the amino acid sequences of the third exon in PBPs should possess three conserved cysteine amino acids (Willett & Harrison, 1999). Hence, the identified MvitPBPs are of the expected size with the presence of six highly conserved cysteine residues.

Pheromone‐binding proteins have six α‐helices with the pheromone ligand bound in an internal hydrophobic pocket (Sandler et al., 2000). Subsequent studies revealed a seventh α‐helix, formed from the C‐terminal tail (Horst et al., 2001). We ascertained the location of seven α‐helices in MvitPBPs by aligning their amino acid sequences with PBPs and OBPs from Bombycidae, Saturniidae, Sphingidae, and Noctuidae (Malini, 2017). Interestingly, these locations were almost similar to the seven α‐helices identified for *B. mori* and *L. dispar* (Yu et al., 2012). The three disulfide bonds in MvitPBPs are the same as the two that attach α3 to helices α1 and α6 (Cys19–Cys54 and Cys50–Cys108, but Cys50–Cys109 for MvitPBP3), and the third disulfide bond (Cys97–Cys117 but Cys98–Cys118 for MvitPBP3) connecting helices α5 and α6 reported in *B. mori* (Sandler et al., 2000). Met74 in α4 and Ile91 in α5 of *B. mori* PBP were substituted by Gln74 and Val91, respectively, in MvitPBP1. Although Met74 was not substituted by another hydrophobic amino acid, Ile91 was substituted by the hydrophobic amino acid. The amino acids of helices α5 and α6 used to form a hydrophobic assembly in *B. mori* (Sandler et al., 2000) are the same in MvitPBP1 except Ile93 in α5, which was replaced by hydrophobic Leu93. In other small interhelix contacts, especially between helices α2 and α3, three substitutions (Val48Thr, Leu52Ile and Met55Leu) were found in MvitPBP1. A loop formed by amino acid residues 60–69 is the most flexible region of the protein, and it serves as the lid into the pheromone‐binding pocket (Nemoto, Uebayasi, & Komeiji, 2002). Thus, the identified MvitPBPs are similar to the structure of already reported lepidopteran PBPs or GOBPs. Although structural modeling was used to predict the “presumed” structures of MvitPBPs (Mao et al., 2016), future studies should confirm their three‐dimensional structures by X‐ray diffraction and/or NMR spectroscopy.

The current study confirmed that MvitPBP1 amino acid sequence was quite similar to most reported PBPs/GOBPs. However, His74 in South America *Maruca* populations was similar to *C. punctiferalis, C. medinalis, Ostrinia nubilalis, O. furnacalis, O. latipenis,* and *L. sticticalis*, whereas it was Gln74 in other *Maruca* populations. Since histidine (His) is involved in pH‐dependent conformational change (Liu, Liu, & Dong, 2013). His74 could induce the conformational change in MvitPBP1 in South American *Maruca* populations. Positively charged His74 in South American populations instead of uncharged Gln74 in other populations may also impact the hydrophobicity and thus affecting the pheromone binding. In addition, interaction of His residues with α6 helix is believed to play a role in loop destabilization and pheromone access to the binding pocket (Lautenschlager, Leal, & Clardy, 2005). Substitution of polar Gln47 in South American (also in few Southeast Asian) populations instead of nonpolar Leu47 in other populations could affect the interhelix contact between α2 and α3 helices. MvitPBP2 differed from most reported PBPs/GOBPs by at least six amino acid substitutions. Although most of these amino acids were predicted not to be located in the pheromone‐binding pocket, Leu94 is expected to be located in or near the hydrophobic‐binding pocket. In addition, Lys70 might induce a conformational change. Alanine–threonine interchange was found in the 74th and 102nd position of the African *M. vitrata* populations, which could differentiate it from other populations since all alanine residues are conserved in lepidopteran PBPs (Sandler et al., 2000). It should also be noted that most of the residues lining the binding pockets were hydrophobic. However, hydrophilic residues, such as threonine present in the binding sites, are probably responsible for hydrogen bonding with the functional group of the ligand (Yu et al., 2012). Hence, the effects of the replacement of alanine by threonine should be thoroughly investigated in subsequent studies. However, because of the hydrophobic nature, both Leu109Met and Leu110Val interchanges may not be of practical significance in MvitPBP2 although they are fixed in all African populations. For MvitPBP3, Gln66 substitution instead of Glu66 in South American *Maruca* and in one PNG populations is quite important, because Glu66 formed H‐bond with the pheromone compound, E10‐16: OH (Mao et al., 2016). Hence, it is possible that some of the identified polymorphisms may be involved in interactions between the PBP and other signal transduction system components including pheromone receptor as reported earlier (Newcomb et al., 2002; Prestwich & Du, 1997; Rogers, Sun, Lerner, & Vogt, 1997). Thus, this warrants further detailed studies to understand whether these polymorphisms contribute toward the reported differential pheromone recognition patterns of male *M. vitrata* moths in different geographical regions (Downham et al., 2004; Schläger et al., 2012; Srinivasan et al., 2015).

Since PBP sequences of samples collected in target countries showed both positive and negative Tajima's *D* values for *MvitPBP1* and *MvitPBP2*, but negative values were obtained for all samples except Vietnam for *MvitPBP3*, we considered continent based Tajima's *D* value for discussion purposes. The negative Tajima's *D* values for Asia, Africa, and Oceania *Maruca* populations for all the PBP genes, and only for *MvitPBP3* for South America indicated the recent expansion of *Maruca* populations, and they provide evidence for purifying selection at this locus. In our earlier study based on *coxI* gene as well (Malini et al., 2015), we found similar results for Asia and Africa. However, the current results for Oceania and South America contradicted our earlier finding. This is possible because *Maruca* populations from Asia and Africa in the current study were similar to the earlier grouping of Asian or African *Maruca* populations. However, the Oceania *Maruca* populations formed two groups—one exclusively in Oceania (including Kalimantan, Indonesia) and the other aligned with Asian and African *M. vitrata* populations in our earlier study (Malini et al., 2015), but not in the current study despite the fact that we collected *Maruca* populations extensively from PNG (especially Madang and Milne Bay) and East Kalimantan (Indonesia), where the other *Maruca* sp. was found earlier. Similarly, we used only the Colombia samples to represent South America in the current study, whereas we had access to several *Maruca coxI* sequences from multiple countries in South America in our earlier study (Malini et al., 2015). Hence, *Maruca* populations in the target continents could have experienced recent demographic expansion events. Apart from the South America populations, all other populations showed negative values for Fu's *F_S_* test with high significance. Although Tajima's *D* values were nonsignificantly negative for most of our *Maruca* populations, Fu's *F_S_* values were significantly negative. This could be due to Fu's *F_S_* being more sensitive in detecting population expansion (Liao et al., 2010). Thus, besides Tajima's *D*, a negative value of Fu's *F_S_* for most of our studied populations is evidence for a possible recent population expansion or genetic drift due to random sampling. Negative values of Fu's *F_S_* are usually caused by an excess of singletons in population expansion events (Fu, 1995, 1997). A positive value of Fu's *F_S_* for South America populations is evidence for the deficiency of alleles due to recent population decline. The South America *Maruca* populations were nonsignificantly positive for both Tajima's *D* (except for *MvitPBP3*) and Fu's *F_S_*. The South America populations were sampled only from Colombia and had a lower sample size, which may not be enough to assess evolutionary neutrality in this region. The statistically highly significant numbers for Fu's *F_S_* indicating recent *Maruca* population growth in Asia, Africa, and Oceania is not confined by local geographical regions (Liao et al., 2010) within a continent. Although *Maruca* populations are speculated to have expanded recently, a large stable population with a long evolutionary history might be the case in Africa, Oceania, and South America, which showed high haplotype and nucleotide diversities for *MvitPBP1*, *MvitPBP2,* and *MvitPBP3*, respectively.

It should be noted that even subspecies could be genetically differentiated and that *F*_ST_ values must be at least 0.25–0.30 for subspecies or races to be recognized (Graves, 2010; Smith, Chiszar, & Montanucci, 1997; Templeton, 1998). Compared with the other populations in the current study, *Maruca* populations from Colombia could be a different putative species of *Maruca* based on *F*_ST_ values (0.41 to 0.73) for all the three PBP genes. Similarly, *Maruca* populations from Kenya and Benin also seemed to be a different subspecies or race on the basis of *F*_ST_ values (0.12–0.73) for all three PBP genes. This was also further supported by the haplotype networks (Figure 9a‐c), in which the African *Maruca* populations mostly formed a separate cluster. However, the *Maruca* adults from Africa and Asia did not show any differences in their morphological characters (Srinivasan et al., 2013), whereas Colombian *Maruca* adults showed some differences in wing characters in a preliminary study (data not shown). This is possible because of recent population expansions and accumulated mutations at the silent sites, which are supported by the negative Tajima's *D* and Fu's *F_S_* values, especially for the Asian, African, and Oceania populations. Thus, it is reasonable to speculate that the Asian and African *Maruca* populations may belong to different subspecies, but the South America populations could be a different *Maruca* species, which should be confirmed by detailed morphological characterization in future studies. Similar results were also obtained in our study based on *coxI* gene for *Maruca* populations from different continents (Malini et al., 2015). It is interesting to note that the Oceania populations in our previous study clearly separated as a different putative *Maruca* species, whereas it was not the case in the current study. We hypothesized the presence of two different *Maruca* species in Oceania (including parts of Indonesia) in our earlier study, but we did not see such a separation based on the *Maruca* samples in this study, although the sampling was done in distant geographical locations at various altitudes (5–1,768 m above sea level) in PNG. However, it should also be noted that the Oceania (Fiji) population did not differ significantly from the Colombia population based on *F*_ST_ values for all three PBP genes, which lead to the speculation that Oceania may still have two different *Maruca* species. Hence, it is proposed to have additional samples collected from Fiji and/or other parts of Oceania (especially islands in the Pacific and Australia, where legume host plants are abundant) for further morphological and molecular characterization.

The phylogenetic analysis clearly differentiated the studied *Maruca* populations from Asia, Africa, Oceania, and South America based on MvitPBPs, despite the striking similarities of MvitPBP1 among the populations. However, the amino acid polymorphisms found in MvitPBP2, specifically the alanine–threonine interchange in the African and other continental *Maruca* populations might have been responsible for the split of these populations into two clades. *Maruca* populations from Colombia formed a separate clade based on both MvitPBP1 and MvitPBP3; although it aligned with the Asian clade in MvitPBP2 based phylogenetic tree, it formed a subclade with a high bootstrap value within the Asian clade. These results indicated the genetic dissimilarity of *Maruca* populations originating from South America with rest of the World populations. These results were further supported by similar results from ABGD analysis, indicating the possible presence of different *Maruca* subspecies in Asia, Africa and Oceania or species in South America.

Allelic variations within PBPs have been reported at both nucleotide and protein levels in previous studies, which might lead to the variations among the individuals of the same species in discriminating different blends of the same pheromone (Newcomb et al., 2002). Some of the populations in the current study resulted in two forms of the same PBP in PAGE, but it was not clear whether they are allelic or products of separate locus. They are present in both Asia and Africa, although the frequency of occurrence is higher for Asia. Further studies to understand the similarities or differences present between these two forms might be useful, because allelic variations could lead to the presence of homozygotes and heterozygotes in field conditions. They might differ in detecting different components of the same pheromone blend, as evidenced in *Epiphyas postvittana* (Newcomb et al., 2002). Hence, future tracking of the frequencies of these forms in natural *Maruca* populations becomes imperative.

## 5 CONCLUSIONS

The moths of *M. vitrata* express three PBPs (MvitPBP1, MvitPBP2, and MvitPBP3), which are structurally similar to earlier reported lepidopteran PBPs. However, MvitPBP2 has at least six amino acid substitutions among the studied populations, including one amino acid residue located in the hydrophobic‐binding pocket. Although alanine residues are conserved in lepidopteran PBPs, alanine–threonine interchanges among the Asian and African populations are observed in two locations of MvitPBP2. These substitutions split the populations into different clades on phylogenetic trees, which are also evidenced from ABGD analysis. Negative Tajima's *D* and Fu's *F_S_* values especially for the Asian, African, and Oceania *Maruca* populations revealed recent population expansions and accumulated mutations in the silent sites. Higher *F*_ST_ values (up to 0.73) for all PBP genes among the studied *Maruca* populations confirmed the presence of different subspecies and/or species in different geographical locations. Thus, the differences in *coxI* sequences among geographically distinct *M. vitrata* populations (Malini et al., 2015) have also been confirmed based on *MvitPBPs*. However, the presence of two different *Maruca* species in Oceania in our earlier study was not confirmed in this study, leading to the speculation that the occurrence of the second *Maruca* species is rare and limited in PNG. The differences in PBPs may also explain the different affinity of African and Asian populations to same pheromone blend(s), because of the presence of different subspecies or races of *M*. *vitrata*. However, future binding studies and elucidation of additional PBPs among various *Maruca* populations in Asia, Africa, Oceania, and South America could shed more light on this perspective, which would also enable to develop pheromone lures specific for a particular *Maruca* population in a given geographical region. Since species‐specific bio‐control agents can provide significant control of a target pest species, the genetic differences among the *Maruca* populations in different geographical regions of the world should also carefully be considered for classical biological control of *Maruca* spp.

## CONFLICT OF INTEREST

None declared.

## AUTHOR CONTRIBUTION

PM: Conceptualize the study, designing of experiments, sample collection, conducting the experiments and data collection, data analysis, manuscript preparation. RS: Conceptualize the study, sample collection, data analysis, Research Grant and Project Management, manuscript revision. RS: Research Supervision, support for data analysis, manuscript review and revision. KM: Research Supervision, Review of research results, manuscript review and revision.

## Data Availability

The data that support the findings of this study are deposited in NCBI GenBank (*MvitPBP1*: MK548942–MK549033, *MvitPBP2*: MK549034–MK549121, *MvitPBP3*: MK561786–MK561853).

## APPENDIX 1

### List of identified *Maruca vitrata* PBP1 haplotypes with their geographical origin and host plants

**Table:**

Haplotype	Representative Sample	Haplotype frequency	Population(s)	NCBI accession number
1	SW‐Transcript	1	Taiwan (*Sesbania cannabina*)	MK548944
2	FA3, FA1	2	Colombia (*Dioclea guianensis*)	MK548942
3	FA4, FA2	2	Colombia (*D. guianensis*)	MK548943
4	XG10	1	PNG (*Vigna unguiculata* subsp. *sesquipedalis*)	MK548945
5	TG1, TG2	2	PNG (*Tephrosia candida*)	MK548946
6	TGK4	1	PNG (*T. candida*)	MK548947
7	TGK5	1	PNG (*T. candida*)	MK548948
8	TGT2	1	PNG (*T. candida*)	MK548949
9	EG3	1	PNG (*Pueraria phaseoloides*)	MK548950
10	EG6	1	PNG (*P. phaseoloides*)	MK548951
11	EGW9	1	PNG (*P. phaseoloides*)	MK548952
12	VG5	1	PNG (*V. unguiculata* subsp. *sesquipedalis*)	MK548953
13	VG8	1	PNG (*V. unguiculata* subsp. *sesquipedalis*)	MK548954
14	VGA3	1	PNG (*V. unguiculata* subsp. *sesquipedalis*)	MK548955
15	VGA5	1	PNG (*V. unguiculata* subsp. *sesquipedalis*)	MK548956
16	VGB4	1	PNG (*V. unguiculata* subsp. *sesquipedalis*)	MK548957
17	VGG5	1	PNG (*V. unguiculata* subsp. *sesquipedalis*)	MK548958
18	VGW6	1	PNG (*V. unguiculata* subsp. *sesquipedalis*)	MK548959
19	YG2	1	PNG (*Dolichos lablab*)	MK548960
20	YGG1	1	PNG (*D. lablab*)	MK548961
21	VF2	1	Fiji (*V. unguiculata* subsp. *sesquipedalis*)	MK548962
22	IMP1	1	Malaysia (*Vigna sinensis*)	MK548963
23	OMK1	1	Malaysia (*Phaseolus vulgaris*)	MK548964
24	OMK4	1	Malaysia (*P. vulgaris*)	MK548965
25	OMK5	1	Malaysia (*P. vulgaris*)	MK548966
26	OMR1	1	Malaysia (*P. vulgaris*)	MK548967
27	PM1	1	Malaysia (*Cajanus cajan*)	MK548968
28	VM3	1	Malaysia (*V. unguiculata* subsp. *sesquipedalis*)	MK548969
29	IMG1	1	Malaysia (*V. sinensis*)	MK548970
30	IMS5	1	Malaysia (*V. sinensis*)	MK548971
31	IMS9	1	Malaysia (*V. sinensis*)	MK548972
32	IMS10	1	Malaysia (*V. sinensis*)	MK548973
33	XM6	1	Malaysia ()	MK548974
34	OMC6	1	Malaysia (*P. vulgaris*)	MK548975
35	IM1B	1	Malaysia (*V. sinensis*)	MK548976
36	IM2B	1	Malaysia (*V. sinensis*)	MK548977
37	IM1T	1	Malaysia (*V. sinensis*)	MK548978
38	IM2T	1	Malaysia (*V. sinensis*)	MK548979
39	IMU9	1	Malaysia (*V. sinensis*)	MK548980
40	VNK8	1	Indonesia (*V. unguiculata* subsp. *sesquipedalis*)	MK548981
41	VNK10	1	Indonesia (*V. unguiculata* subsp. *sesquipedalis*)	MK548982
42	CN12	1	Indonesia (*Vigna unguiculata*)	MK548983
43	GN5, VT1, VT5	3	Indonesia (*Sesbania grandiflora*) Thailand (*V. unguiculata* subsp. *sesquipedalis*)	MK548984
44	HL4	1	Laos (*Phaseolus* sp.)	MK548985
45	HL7	1	Laos (*Phaseolus* sp.)	MK548986
46	ML4	1	Laos (*Vigna radiata*)	MK548987
47	VLH2	1	Laos (*V. unguiculata* subsp. *sesquipedalis*)	MK548988
48	VLN4	1	Laos (*V. unguiculata* subsp. *sesquipedalis*)	MK548989
49	VLN7	1	Laos (*V. unguiculata* subsp. *sesquipedalis*)	MK548990
50	VLV2	1	Laos (*V. unguiculata* subsp. *sesquipedalis*)	MK548991
51	VC6B, VC3	2	Cambodia (*V. unguiculata* subsp. *sesquipedalis*)	MK548992
52	VC9, VCK1	2	Cambodia (*V. unguiculata* subsp. *sesquipedalis*)	MK548993
53	VCK3, VC7	2	Cambodia (*V. unguiculata* subsp. *sesquipedalis*)	MK548994
54	MT3, MT8	2	Thailand (*V. radiata*)	MK548995
55	WT7, WT2	2	Thailand (*Psophocarpus tetragonolobus*)	MK548996
56	BV1	1	Vietnam (*Vigna cylindrica*)	MK548997
57	GV1	1	Vietnam (*S. grandiflora*)	MK548998
58	GV3	1	Vietnam (*S. grandiflora*)	MK548999
59	MVM5	1	Vietnam (*V. radiata*)	MK549000
60	OV14_2	1	Vietnam (*P. vulgaris*)	MK549001
61	OVB2	1	Vietnam (*P. vulgaris*)	MK549002
62	VV5_5	1	Vietnam (*V. unguiculata* subsp. *sesquipedalis*)	MK549003
63	SW5, VVB6B	2	Taiwan (*Sesbania cannabina*) Vietnam (*V. unguiculata* subsp. *sesquipedalis*)	MK549004
64	AW5	1	Taiwan (*Canavalia* sp.)	MK549005
65	CW5	1	Taiwan (*V. unguiculata*)	MK549006
66	GW10	1	Taiwan (*S. grandiflora*)	MK549007
67	MW4	1	Taiwan (*V. radiata*)	MK549008
68	SW3	1	Taiwan (*S. cannabina*)	MK549009
69	VW13	1	Taiwan (*V. unguiculata* subsp. *sesquipedalis*)	MK549010
70	ZW2	1	Taiwan (*Vigna angularis*)	MK549011
71	CD5	1	India (*V. unguiculata*)	MK549012
72	YDR6	1	India (*D. lablab*)	MK549013
73	YDS9	1	India (*D. lablab*)	MK549014
74	CS7	1	Bangladesh (*V. unguiculata*)	MK549015
75	UB1, AB2	2	Benin (*Pterocarpus santalinoides*; *Canavalia* sp.)	MK549016
76	EB2	1	Benin (*P. phaseoloides*)	MK549017
77	LB3	1	Benin (*Lonchocarpus cyanesens*)	MK549018
78	LB9	1	Benin (*L. cyanesens*)	MK549019
79	RB1	1	Benin (*Sesbania rostrata*)	MK549020
80	TB7	1	Benin (*Tephrosia bracteola*)	MK549021
81	TB1	1	Benin (*T. bracteola*)	MK549022
82	UB4	1	Benin (*P. santalinoides*)	MK549023
83	UB5	1	Benin (*P. santalinoides*)	MK549024
84	EK7	1	Kenya (*P. phaseoloides*)	MK549025
85	NK8, OK1	2	Kenya*(Centrosema pubescens*) (*P. vulgaris*)	MK549026
86	NK10	1	Kenya (*C. pubescens*)	MK549027
87	OK8	1	Kenya (*P. vulgaris*)	MK549028
88	OKN9	1	Kenya (*P. vulgaris*)	MK549029
89	OKR6	1	Kenya (*P. vulgaris*)	MK549030
90	PKM7	1	Kenya (*C. cajan*)	MK549031
91	TK9	1	Kenya (*T. bracteola*)	MK549032
92	YK4	1	Kenya (*D. lablab*)	MK549033

## APPENDIX 2

### List of identified *Maruca vitrata* PBP2 haplotypes with their geographical origin and host plants

**Table:**

Haplotype	Representative sample	Haplotype frequency	Population(s)	NCBI accession number
1	SW‐Transcript	1	Taiwan (*Sesbania cannabina*)	MK549036
2	FA3, FA7, QA1, QA6, VF4,	5	Colombia (*Dioclea guianensis*) Colombia (*Dioclea trujillensis*) Fiji (*Vigna unguiculata* subsp. *sesquipedalis*)	MK549034 MK549035 MK549049
3	PG1	1	PNG (*Cajanus cajan*)	MK549037
4	TGY1	1	PNG (*Tephrosia candida*)	MK549038
5	TG3	1	PNG (*T. candida*)	MK549039
6	TG5	1	PNG (*T. candida*)	MK549040
7	EGW10	1	PNG (*P. phaseoloides*)	MK549041
8	VG8	1	PNG (*V. unguiculata* subsp. *sesquipedalis*)	MK549042
9	VGB7	1	PNG (*V. unguiculata* subsp. *sesquipedalis*)	MK549043
10	VGG8	1	PNG (*V. unguiculata* subsp. *sesquipedalis*)	MK549044
11	YGG1	1	PNG (*Dolichos lablab*)	MK549045
12	YGG4	1	PNG (*D. lablab*)	MK549046
13	VF2, IMP1	2	Fiji (*V. unguiculata* subsp. *sesquipedalis*) Malaysia (*Vigna sinensis*)	MK549047 MK549050
14	VF3	1	Fiji (*V. unguiculata* subsp. *sesquipedalis*)	MK549048
15	OMK2	1	Malaysia (*Phaseolus vulgaris*)	MK549051
16	OMK4, IMS5	2	Malaysia (*P. vulgaris*; *V. sinensis*)	MK549052 MK549057
17	VM3, PW4	2	Malaysia (*V. unguiculata* subsp. *sesquipedalis*) Taiwan (*C. cajan*)	MK549053 MK549080
18	OM2	1	Malaysia (*P. vulgaris*)	MK549054
19	OMC6	1	Malaysia (*P. vulgaris*)	MK549055
20	XM6	1	Malaysia (*V. unguiculata* subsp. *sesquipedalis*)	MK549056
21	IMS9	1	Malaysia (*V. sinensis*)	MK549058
22	VNK8, VNK1	2	Indonesia (*V. unguiculata* subsp. *sesquipedalis*)	MK549059
23	VNK10, VNK3	2	Indonesia (*V. unguiculata* subsp. *sesquipedalis*)	MK549060
24	VCK1, VC2	2	Cambodia (*V. unguiculata* subsp. *sesquipedalis*)	MK549061
25	VCK2, VCK7	2	Cambodia (*V. unguiculata* subsp. *sesquipedalis*)	MK549062
26	VCK3, VC8	2	Cambodia (*V. unguiculata* subsp. *sesquipedalis*)	MK549063
27	GT5	1	Thailand (*Sesbania grandiflora*)	MK549064
28	VT1	1	Thailand (*V. unguiculata* subsp. *sesquipedalis*)	MK549065
29	WT3	1	Thailand (*Psophocarpus tetragonolobus*)	MK549066
30	WT7	1	Thailand (*P. tetragonolobus*)	MK549067
31	ML4, DL3	2	Laos (*Vigna radiata; Sesbania vesicaria*)	MK549068
32	PL4, VLS3	2	Laos (*C. cajan*; *V. unguiculata* subsp. *Sesquipedalis*)	MK549069
33	VLH2, VLV5	2	Laos (*V. unguiculata* subsp. *sesquipedalis*)	MK549070
34	GV3	1	Vietnam (*S. grandiflora*)	MK549071
35	GV4	1	Vietnam (*S. grandiflora*)	MK549072
36	MVM1	1	Vietnam (*V. radiata*)	MK549073
37	MVM5	1	Vietnam (*V. radiata*)	MK549074
38	OVB2	1	Vietnam (*P. vulgaris*)	MK549075
39	VV142	1	Vietnam (*V. unguiculata* subsp. *sesquipedalis*)	MK549076
40	AW5	1	Taiwan (*Canavalia* sp.)	MK549077
41	CW2	1	Taiwan (*Vigna unguiculata*)	MK549078
42	MW4	1	Taiwan (*V. radiata*)	MK549079
43	SW5	1	Taiwan (*S. cannabina*)	MK549081
44	VW13	1	Taiwan (*V. unguiculata* subsp. *sesquipedalis*)	MK549082
45	YW6	1	Taiwan (*D. lablab*)	MK549083
46	CS3	1	Bangladesh (*V. unguiculata*)	MK549084
47	CS7	1	Bangladesh (*V. unguiculata*)	MK549085
48	CD5	1	India (*V. unguiculata*)	MK549086
49	YDR3	1	India (*D. lablab*)	MK549087
50	YDS9	1	India (*D. lablab*)	MK549088
51	AB1	1	Benin (*Canavalia* sp.)	MK549089
52	AB2	1	Benin (*Canavalia* sp.)	MK549090
53	AB3	1	Benin (*Canavalia* sp.)	MK549091
54	AB10	1	Benin (*Canavalia* sp.)	MK549092
55	CB4	1	Benin (*V. unguiculata*)	MK549093
56	CB9	1	Benin (*V. unguiculata*)	MK549094
57	EB2	1	Benin (*P. phaseoloides*)	MK549095
58	EB10	1	Benin (*P. phaseoloides*)	MK549096
59	LB1	1	Benin (*Lonchocarpus cyanesens*)	MK549097
60	LB9	1	Benin (*L. cyanesens*)	MK549098
61	RB9	1	Benin (*Sesbania rostrata*)	MK549099
62	RB10	1	Benin (*S. rostrata*)	MK549100
63	TB1	1	Benin (*Tephrosia bracteola*)	MK549101
64	TB2	1	Benin (*T. bracteola*)	MK549102
65	TB3	1	Benin (*T. bracteola*)	MK549103
66	TB5	1	Benin (*T. bracteola*)	MK549104
67	UB1	1	Benin (*Pterocarpus santalinoides*)	MK549105
68	UB5	1	Benin (*P. santalinoides*)	MK549106
69	CKS3, CKO2, OKN8	3	Kenya (*V. unguiculata*; *P. vulgaris*)	MK549107 MK549108 MK549115
70	EK5	1	Kenya (*P. phaseoloides*)	MK549109
71	EK8	1	Kenya (*P. phaseoloides*)	MK549110
72	JK2, OKR6, PKM7	3	Kenya (*Crotalaria juncea; P. vulgaris*; *C. cajan*)	MK549111 MK549116 MK549118
73	NK8, OK8, OKN5	3	Kenya (*Centrosema pubescens; P. vulgaris*)	MK549112–MK549114
74	PKM5	1	Kenya (*C. cajan*)	MK549117
75	TK6	1	Kenya (*T. bracteola*)	MK549119
76	YK1	1	Kenya (*D. lablab*)	MK549120
77	YK4	1	Kenya (*D. lablab*)	MK549121

## APPENDIX 3

### List of identified *Maruca vitrata* PBP3 haplotypes with their geographical origin and host plants

**Table:**

Haplotype	Representative Sample	Haplotype frequency	Population(s)	NCBI accession number
1	SW‐Transcript	1	Taiwan (*Sesbania cannabina*)	MK561791
2	FA3	1	Colombia (*Dioclea guianensis*)	MK561786
3	FA4	1	Colombia (*Dioclea guianensis*)	MK561787
4	FA6	1	Colombia (*Dioclea guianensis*)	MK561788
5	FA8	1	Colombia (*Dioclea guianensis*)	MK561789
6	QA1	1	Colombia (*T. candida*)	MK561790
7	PG1	1	PNG (*Cajanus cajan*)	MK561794
8	TGY1	1	PNG (*Tephrosia candida*)	MK561795
9	EG5	1	PNG (*P. santalinoides*)	MK561792
10	EGW9	1	PNG (*P. santalinoides*)	MK561793
11	VG5	1	PNG (*V. unguiculata* subsp. *sesquipedalis*)	MK561796
12	VGG1	1	PNG (*V. unguiculata* subsp. *sesquipedalis*)	MK561800
13	VGG5	1	PNG (*V. unguiculata* subsp. *sesquipedalis*)	MK561801
14	VGB7	1	PNG (*V. unguiculata* subsp. *sesquipedalis*)	MK561799
15	VGA1	1	PNG (*V. unguiculata* subsp. *sesquipedalis*)	MK561797
16	VGA5	1	PNG (*V. unguiculata* subsp. *sesquipedalis*)	MK561798
17	YGG1	1	PNG (*Dolichos lablab*)	MK561802
18	YGG4	1	PNG (*D. lablab*)	MK561803
19	VF1	1	Fiji (*V. unguiculata* subsp. *sesquipedalis*)	MK561804
20	IMP1	1	Malaysia (*Vigna sinensis*)	MK561805
21	OMK4	1	Malaysia (*Phaseolus vulgaris*)	MK561808
22	VM3	1	Malaysia (*V. unguiculata* subsp. *sesquipedalis*)	MK561809
23	IMS5	1	Malaysia (*V. sinensis*)	MK561806
24	IMU9	1	Malaysia (*V. sinensis*)	MK561807
25	XM6	1	Malaysia (*V. unguiculata* subsp. *sesquipedalis*)	MK561810
26	VNK2	1	Indonesia (*V. unguiculata* subsp. *sesquipedalis*)	MK561811
27	VNK8	1	Indonesia (*V. unguiculata* subsp. *sesquipedalis*)	MK561812
28	VNK9	1	Indonesia (*V. unguiculata* subsp. *sesquipedalis*)	MK561813
29	VNK10	1	Indonesia (*V. unguiculata* subsp. *sesquipedalis*)	MK561814
30	DL1T	1	Laos (*Sesbania vesicaria*)	MK561815
31	DL1B	1	Laos (*S. vesicaria*)	MK561816
32	HL7	1	Laos (*Phaseolus* sp.)	MK561817
33	PL1	1	Laos (*V. radiata*)	MK561818
34	VLH2	1	Laos (*V. unguiculata* subsp. *sesquipedalis*)	MK561819
35	BV1, BV2	2	Vietnam (*Vigna cylindrica*)	MK561820, MK561821
36	GV4	1	Vietnam (*Sesbania grandiflora*)	MK561822
37	MVM5	1	Vietnam (*V. radiata*)	MK561823
38	CS3	1	Bangladesh (*V. unguiculata*)	MK561831
39	CS7	1	Bangladesh (*V. unguiculata*)	MK561832
40	CD5	1	India (*V. unguiculata*)	MK561828
41	YDR1	1	India (*D. lablab*)	MK561829
42	YDS9	1	India (*D. lablab*)	MK561830
43	GT5	1	Thailand (*S. grandiflora*)	MK561824
44	MT3T	1	Thailand (*V. radiata*)	MK561826
45	MT3B	1	Thailand (*V. radiata*)	MK561825
46	WT7	1	Thailand (*Psophocarpus tetragonolobus*)	MK561827
47	MW4	1	Taiwan (*V. radiata*)	MK561833
48	PW4	1	Taiwan (*C. cajan*)	MK561834
49	SW5	1	Taiwan (*S. cannabina*)	MK561835
50	VW1	1	Taiwan (*V. unguiculata* subsp. *sesquipedalis*)	MK561836
51	AB3	1	Benin (*Canavalia* sp.)	MK561837
52	CB4	1	Benin (*V. unguiculata*)	MK561838
53	EB2	1	Benin (*P. phaseoloides*)	MK561839
54	LB5	1	Benin (*Lonchocarpus cyanesens*)	MK561840
55	UB5	1	Benin (*P. santalinoides*)	MK561842
56	RB1	1	Benin (*Sesbania rostrata*)	MK561841
57	EK5	1	Kenya (*P. phaseoloides*)	MK561843
58	JK1	1	Kenya (*Crotalaria juncea*)	MK561844
59	JK9, NK10, PKM2, YK4	4	Kenya (*C. juncea; C. pubescens; C. cajan; D. lablab*)	MK561845, MK561847, MK561851, MK561853
60	NK8	1	Kenya (*C. pubescens*)	MK561846
61	OK1	1	Kenya (*P. vulgaris*)	MK561848
62	OKR6	1	Kenya (*P. vulgaris*)	MK561849
63	PK2	1	Kenya (*C. cajan*)	MK561850
64	TK6	1	Kenya (*T. bracteola*)	MK561852
